# mTORC1/autophagy-regulated MerTK in mutant BRAFV600 melanoma with acquired resistance to BRAF inhibition

**DOI:** 10.18632/oncotarget.18213

**Published:** 2017-05-25

**Authors:** Gongda Xue, Reto Kohler, Fengyuan Tang, Debby Hynx, Yuhua Wang, Francesca Orso, Vincent Prêtre, Reto Ritschard, Petra Hirschmann, Peter Cron, Tim Roloff, Reinhard Dummer, Mario Mandalà, Sandrine Bichet, Christel Genoud, Alexandra G. Meyer, Manuele G. Muraro, Giulio C. Spagnoli, Daniela Taverna, Curzio Rüegg, Taha Merghoub, Daniela Massi, Huifang Tang, Mitchell P. Levesque, Stephan Dirnhofer, Alfred Zippelius, Brian A. Hemmings, Andreas Wicki

**Affiliations:** ^1^ Department of Mechanisms of Cancer, Friedrich Miescher Institute for Biomedical Research, Basel, Switzerland; ^2^ Department of Biomedicine, University Hospital Basel, Basel, Switzerland; ^3^ Molecular Biotechnology Center and Department of Molecular Biotechnology and Health Sciences, University of Torino, Torino, Italy; ^4^ Institute of Pathology, University of Basel, Basel, Switzerland; ^5^ Department of Dermatology, University Hospital of Zurich, Zurich, Switzerland; ^6^ Unit of Clinical and Translational Research, Department of Oncology and Hematology, Papa Giovanni XXIII Hospital, Bergamo, Italy; ^7^ Department of Medicine, University of Fribourg, Fribourg, Switzerland; ^8^ Ludwig Center for Cancer Immunotherapy, Memorial Sloan-Kettering Cancer Center, New York, NY, USA; ^9^ Division of Pathological Anatomy, Department of Surgery and Translational Medicine, University of Florence, Florence, Italy; ^10^ Department of Pharmacology, Zhejiang University, School of Basic Medical Sciences, Hangzhou, China

**Keywords:** Mer tyrosine kinase, drug resistance, BRAF mutation, Zeb2, autophagy

## Abstract

BRAF inhibitors (BRAFi) and the combination therapy of BRAF and MEK inhibitors (MEKi) were recently approved for therapy of metastatic melanomas harbouring the oncogenic BRAFV600 mutation. Although these therapies have shown pronounced therapeutic efficacy, the limited durability of the response indicates an acquired drug resistance that still remains mechanistically poorly understood at the molecular level. We conducted transcriptome gene profiling in BRAFi-treated melanoma cells and identified that Mer tyrosine kinase (MerTK) is specifically upregulated. MerTK overexpression was demonstrated not only in melanomas resistant to BRAFi monotherapy (5 out of 10 samples from melanoma patients) but also in melanoma resistant to BRAFi+MEKi (1 out of 3), although MEKi alone does not affect MerTK. Mechanistically, BRAFi-induced activation of Zeb2 stimulates MerTK in BRAFV600 melanoma through mTORC1-triggered activation of autophagy. Co-targeting MerTK and BRAFV600 significantly reduced tumour burden in xenografted mice, which was pheno-copied by co-inhibition of autophagy and mutant BRAFV600.

## INTRODUCTION

Melanoma is one of only three aggressive cancers whose incidence and mortality have increased continuously over the past two decades [1]. Compared with the commonly used chemotherapeutic agent dacarbazine, treatment with a BRAFi or a BRAFi/MEKi combination results in improved overall survival of patients with metastatic melanoma carrying mutant BRAF [2-5]. However, the durability of response is limited [6] and tumour progression occurs after 6-7 months of monotherapy or around 9 months of combination therapy, indicating the development of compensatory mechanisms that antagonize mutant BRAF and MEK inhibition. When bypassing BRAF^V600E^ activity, resistant melanomas often exhibit reactivation of the interconnected MAPK and PI3K/Akt axes, associated with BRAF-independent reactivation of MAPK [7] (caused in some cases by enhanced CRAF [8]), elevated PDGFR [9], and remodeling of the cancer stroma [10]. However, the molecular mechanisms of resistance are not yet fully understood and need further investigation.

Autophagy is a conserved intracellular degradation system that is well known to be activated downstream of mTORC1 [11, 12]. Enhanced autophagic activity is required for energy metabolism during development. Upon nutrient withdrawal, newborn mice die within one day if autophagic activity is inhibited [13], indicating a pivotal role for autophagy in response to stress cues. Indeed, autophagic activity is elevated in different types of cancer and is considered a therapeutic target in several clinical trials [14, 15]. Nevertheless, the role of autophagy in tumor development and progression is controversial, since a range of studies has hinted at a role of autophagy in promoting apoptosis [16, 17]. These observations support the concept of context-dependent regulatory mechanisms of autophagy during cancer progression [18], possibly through differential regulation of distinct, yet unknown, downstream targets. To dissect the underlying molecular mechanisms of adapted reactivation of the RTK/MAPK signaling in response to BRAF and combined BRAF/MEK inhibition, we conducted transcriptome gene profiling in vemurafenib-treated melanoma cells. We focused on proto-oncogenes encoding kinases that are often hyperactivated in cancers. We confirmed the results in cell lines and clinical melanoma samples that are resistant to BRAFi and BRAFi/MEKi.

Here we show that BRAFi induces upregulation of MerTK, a master regulator of phagocytosis, which contributes to acquired resistance to BRAF inhibition in BRAF^V600E^ melanoma. Our data identify MerTK as a novel mediator of cancer cell survival driven by mTORC1/autophagy signaling, and suggest that combinatorial inhibition of BRAF^V600E^ and the autophagy/MerTK axis provides a novel therapeutic strategy to overcome acquired resistance to BRAFi or BRAFi/MEKi in melanoma patients.

## RESULTS

### MerTK is over-expressed in human melanomas with acquired resistance to vemurafenib

**Figure 1:**
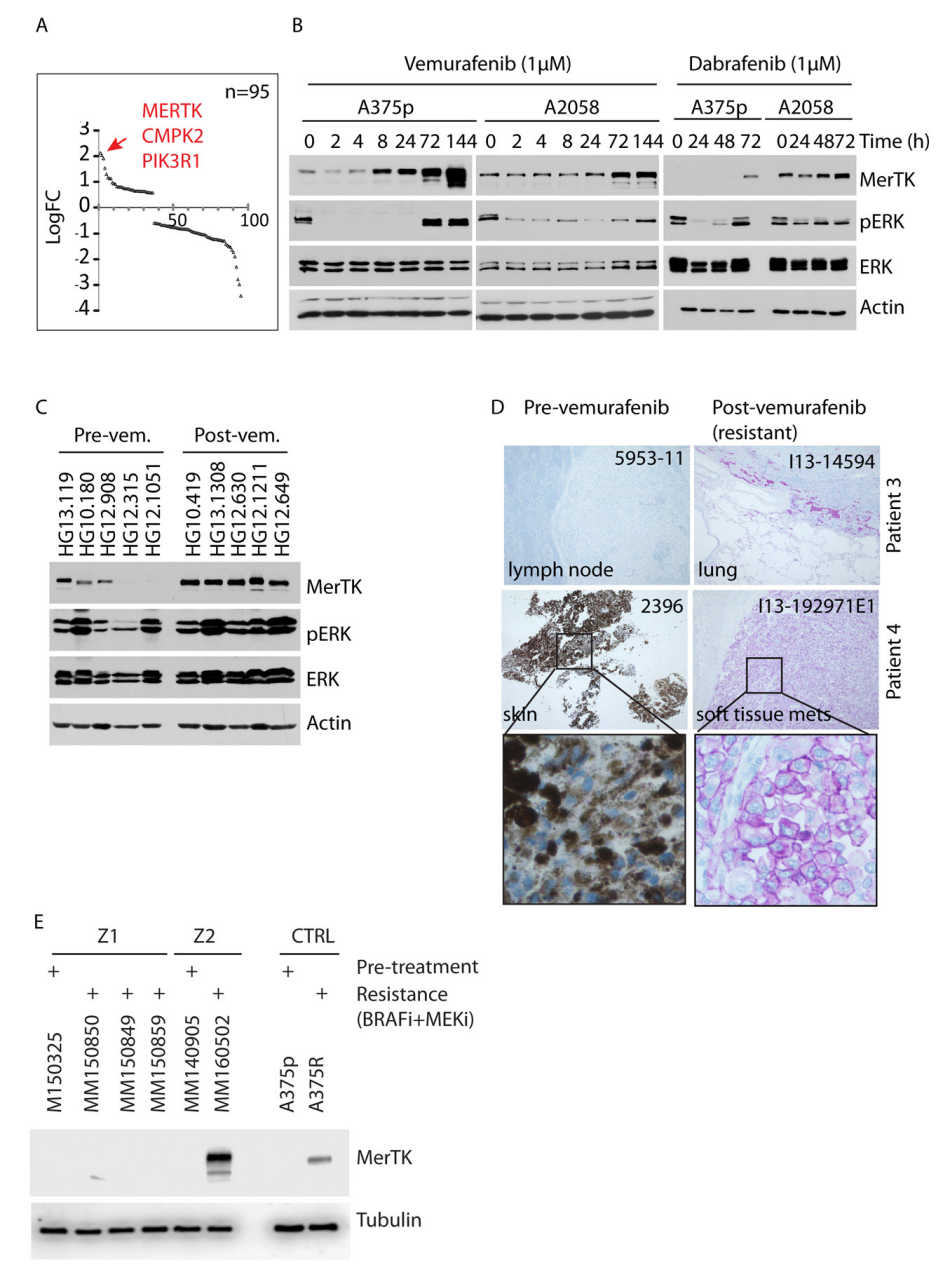
Vemurafenib triggers oncogenic MerTK upregulation in BRAF^V600E^ melanoma cells **A.**, Transcriptome profiling of deregulated kinase-coding genes in melanoma cells treated with vemurafenib (1 μM) for 3 days. 95 kinase-coding genes with significant change (cutoff: *P* = 0.05, linFC = 1.5, average expression = 4) are presented. The red arrow points at the top three genes with highest enrichment. **B.**, A375p and A2058 melanoma cells were treated with 1 μM vemurafenib or dabrafenib and MerTK expression was monitored at the given time-points. The impact on MAPK signaling was determined by the status of pERK (See also Figures S1C, S1D, S1E). **C.**, MerTK expression was examined in 10 human melanomas with pre-/post-vemurafenib therapy (5 of each, unpaired samples; uncropped gel see Figure S1H). 60 μg of total protein was loaded per well. The code of each sample was provided by the University hospital of Zurich. **D.**, Endogenous MerTK expression level was monitored in 2 melanoma patients with pre-/post-vemurafenib therapy (paired samples, diagnosed as resistant melanomas to vemurafenib in clinic) by IHC (See also Figure S1I). The code of each tumor (up-right) was provided by the Papa Giovanni XXIII Hospital (for retrospective study) and the host tissue/organ was indicated (down-left). Purple staining indicates MerTK and light/dark brown indicates melanin (patient 4). Under vemurafenib therapy, patient 4 developed a metastatic tumor (I13-192971E1) of the soft tissue (pathological scoring is shown in supplemental table S2.1 and the clinical characteristics of the patients are shown in supplemental table S2.2). **E.**, Melanoma cells were isolated from Z1 and Z2 melanomas resistant to BRAFi+MEKi therapy. The isolated cancer cells were cultured for one week and total protein was extracted for western blotting analysis. A375R cell line was used as a control for MerTK upregulation.

To investigate RTK-associated genes that impact on the relief of BRAF^V600E^ inhibition, we performed transcriptome screening of BRAF^V600E^ melanoma cells treated with PLX4720, the precursor of vemurafenib (PLX4032) with equivalent action mode [19], for 3 days. Upon completion of therapy, ERK phosphorylation was restored. Gene expression profiling revealed significant upregulation of 95 kinase-encoding genes. The most prominent was MERTK (Figure 1A). Transcriptional upregulation of MERTK and several other genes including CMPK2, PIK3R1, PROS1 and AXL was confirmed by quantitative PCR (qPCR, Supplementary Figure S1A). Therapy-induced overexpression of MerTK on the plasma membrane (Supplementary Figure S1B) was dependent of the time-course and occurred after 48 hours of BRAFi-treatment in 50% (8 out of 16) of the examined BRAF mutant human melanoma cell lines (Figures 1B, S1C and S1D). Similarly, MerTK expression could also be regulated in a dose-dependent manner (Supplementary Figure S1E). BRAFi-triggered MerTK overexpression was specific for melanoma cells carrying a BRAF^V600^ mutation but was not observed in the majority of BRAF wild-type cells (Supplementary Figure S1F). Interestingly, the upregulation of MerTK was often associated with restored ERK phosphorylation post-vemurafenib treatment. Indeed, overexpressed MerTK strongly enhanced its tyrosine phosphorylation, indicating a functional MerTK signaling transduction that might be switched on (Supplementary Figure S1G). MerTK was recently proposed as a potential therapeutic target in melanoma [20]. Compared with untreated human melanomas, increased expression of MerTK was detected in the tumours from vemurafenib-treated patients (Figures 1C, S1H). More importantly, in a comparison between pre- and post-therapy of vemurafenib in the same patient (paired samples), significantly higher MerTK levels were detected in the new lesions after therapy in 50% (5 out of 10) of the patients with clinically diagnosed acquired resistance (Figures 1D, S1I). Moreover, although melanoma cell lines resistant to single-agent MEKi did not upregulate MerTK (Supplementary Figure S1J), ∼30% (1 out of 3) paired human melanomas with acquired resistance to combined BRAFi+MEKi therapy showed elevated MerTK expression (Figures 1E, supplemental Table S2.1). Basal MerTK was exclusively detected in melanomas but not in benign melanocytic nevi (Supplementary Figure S1K). These observations imply a potential role of MerTK in supporting cell survival in malignant melanoma in response to stress.

### MerTK promotes melanoma cell survival and colony formation in response to BRAFi

**Figure 2:**
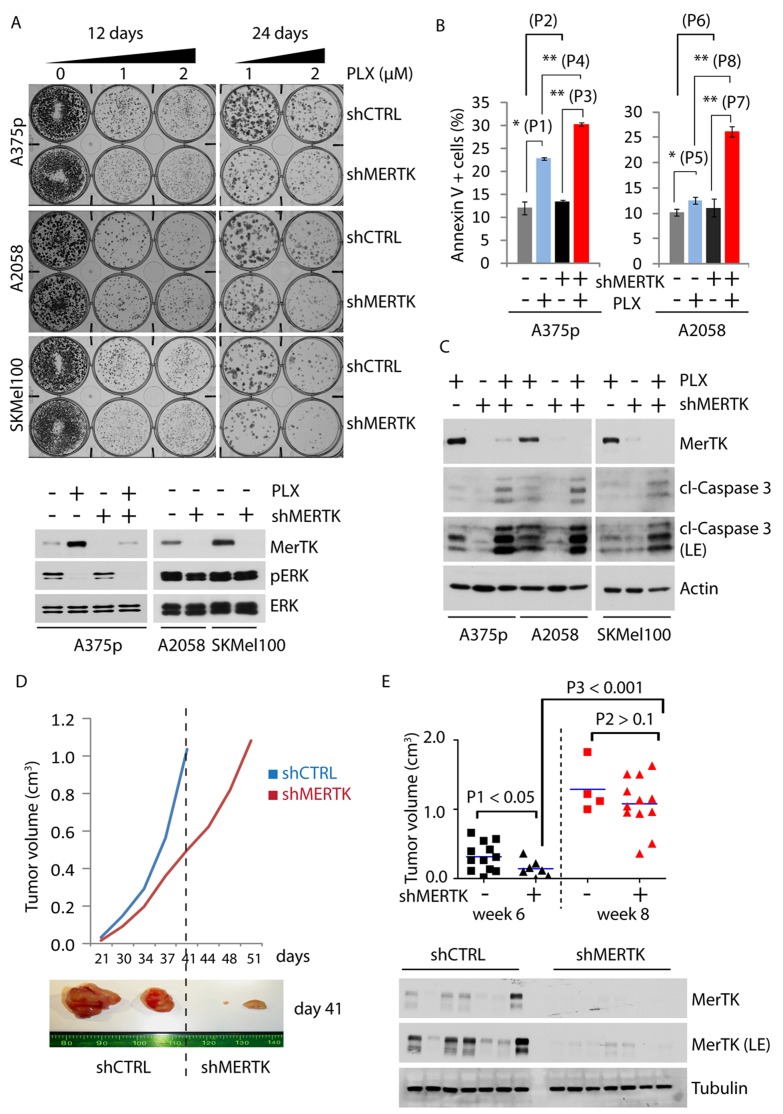
MerTK promotes melanoma cell survival and tumor formation **A.**, Endogenous MERTK was knocked down by shRNA in three melanoma cell lines (expressing BRAF^V600E^ and different basal level of MerTK: A375p, low; A2058, medium; SKMel100, high). Optimization of shRNA was performed (Figure S2F). 500 cells per well were grown on 6-well plates incubated with vemurafenib (1 or 2 μM, equal volume of DMSO was used as control) for 12 or 24 days and stained with crystal violet. The control of MerTK depletion was shown by western blot. The size of the colony was measured with ImageJ (see also Figure S2H). Each sample was prepared in triplicates. **B.**, FACS analysis of apoptotic potential (represented by Annexin V-positive cells) of A375p and A2058 cells with depleted MerTK in presence/absence of vemurafenib. *P*1 = 0.004; *P*2 = 0.227; *P*3 = 4.43E-06; *P*4 = 1.8E-05; *P*5 = 0.015; *P*6 = 0.482; *P*7 = 0.6E-03; *P*8 = 1.5E-04. **C.**, A375p, A2058 and SKMel100 cells with MerTK-depletion were treated with vemurafenib (1 μM) for 3 days and the level of cleaved caspase 3 was determined. **D.**, Growth of xenograft tumours derived from inoculated A375p melanoma cells with MERTK knockdown. 16∼18 mice per group were subcutaneously injected with A375p_shCTRL or A375p_shMERTK. The tumour size was measured twice a week. The resected xenograft tumours represented the size on day 41(week 6 post-injection). **E.**, Statistical comparison of tumour size between A375p_shCTRL (square) or A375p_shMERTK (triangle) at two time points: week 6 and week 8. Depletion of MerTK in the tumors was demonstrated by western blot. P value was calculated with GraphPad Prism.

Resistance against BRAFi develops rapidly in melanoma patients and is characterized by ERK reactivation that promotes cancer cell proliferation and induces a protective response to stress [21]. To investigate whether vemurafenib-elicited MerTK upregulation leads to restoration of proliferation in resistant cell populations, we generated resistant melanoma cell lines A375R and A2058R that were exposed to BRAFi for 2 months. Interestingly, both A375p and A2058 cells underwent continuous morphological change during the treatment. Short-term treatment for 6 days resulted in filopodia-like membrane protrusions with increased actin bundling and enhanced spindle-shaped morphology (Supplementary Figure S2A, white arrow), but the cells gradually became flat (Supplementary Figure S2A, A375R and A2058R) when they lost responsiveness to BRAFi [22] in association with increased MerTK level (Supplementary Figure S2A, red stars). Although the proliferation of PLX-treated cells *in vitro* was restrained when compared with DMSO treatment, the invasive potential was significantly increased in a matrigel-based 3D invasion assay (Supplementary Figure S2B). In fact, untreated A375p cells tended to form aggregates on matrigel, whereas A375R cells were much flatter, displayed individual migratory patterns (Supplementary Figure S2C) and exhibited elevated invasiveness (Supplementary Figure S2D). Notably, MerTK was stably maintained in both resistant cell lines in association with restored ERK and Akt phosphorylation, increased myosin phosphorylation and enhanced expression of fibronectin, the key regulators of cancer cell invasion (Supplementary Figure S2E) [23]. To evaluate the role of MerTK in this phenotype, we decided to study the behavior of the cells upon genetic depletion of MERTK. Knockdown of MERTK with three different shRNAs led to a similar phenotype in A2058 melanoma cells (constitutively expressing MerTK) grown on matrigel (Supplementary Figure S2F), although cell proliferation was not affected (Supplementary Figure S2G). However, when incubated with PLX, the colony formation was severely impaired in three different MERTK-depleted melanoma cell lines A375p, A2058 and SKMel100 (Figures 2A, Supplementary Figure S2H), all of which were associated with an increased pro-apoptotic potential (Figure 2B). Consistently, loss of MerTK sensitized melanoma cells to apoptosis upon BRAF inhibition, as determined by increased accumulation of cleaved Caspase 3 (Figure 2C), suggesting that MerTK is a critical mediator regulating cellular responses in order to antagonize apoptotic stress. This finding was further supported in a xenograft mouse model in which initial tumour formation was significantly delayed after a decrease in MerTK (Figure 2D), although these tumours relapsed later (Figure 2E).

### MerTK is stringently regulated by the autophagic signaling pathway

**Figure 3:**
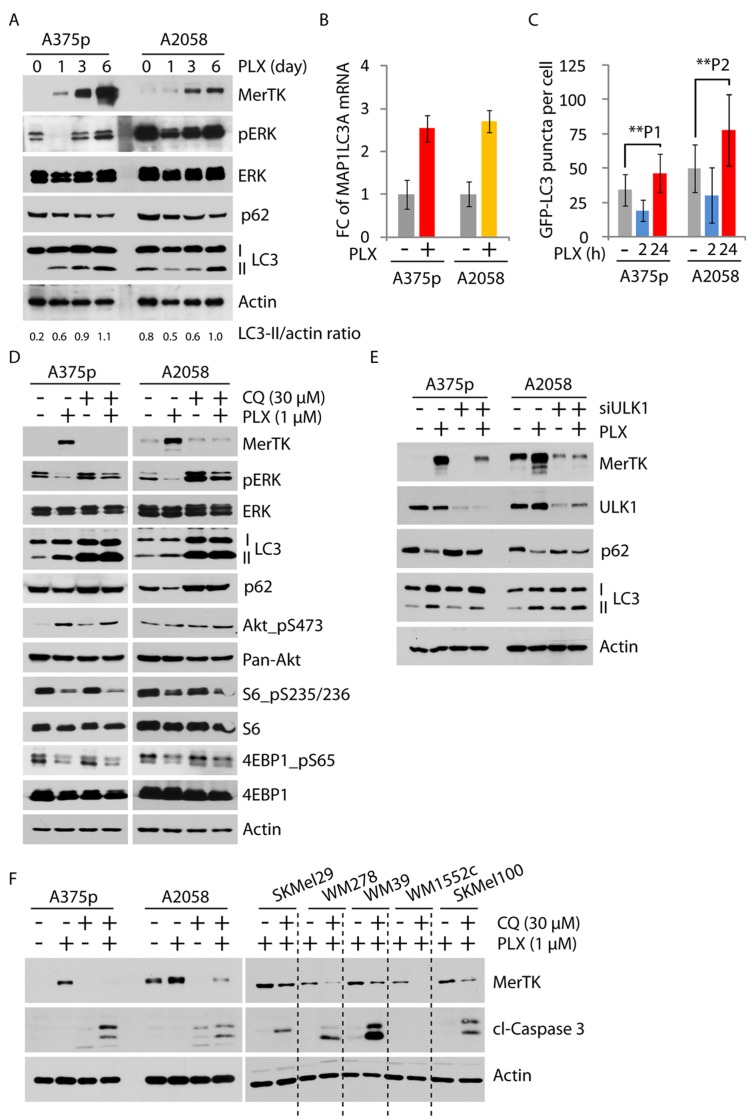
MerTK was stringently regulated by autophagy **A.**, The elevated autophagic activity following vemurafenib treatment (1 μM) was determined by increased LC3 cleavage and enhanced degradation of p62 at given time-points. Increased LC3 cleavage was determined by the ratio of LC3-II *versus* actin measured with ImageJ. **B.**, Global increase of MAP1LC3A mRNA (encoding LC3) was measured by qPCR in A375p and A2058 cells treated with vemurafenib (1 μM) for 3 days. Each sample was measured in triplicates. **C.**, GFP-LC3 puncta (representing autophagosomes) was determined in A375p and A2058 cells expressing GFP-LC3 treated with vemurafenib (1 μM) at indicated time-points. The number of GFP-labeled autophagosomes was counted under the microscope. Three independent transfection experiments were performed. *P*1 = 0.004; *P*2 = 0.0002. See also Figure S3B. **D.**, Expression of MerTK and mTORC1 activity were investigated in melanoma cells treated with vemurafenib (1 μM, 3 days) and the pharmacological autophagy inhibitor Chloroquine (CQ, 30 μM, 3 days). Enriched LC3II fragments indicated defective lysosome-mediated degradation by CQ treatment. **E.**, Determination of MerTK expression level in melanoma cells with transient depletion of autophagy initiating kinase ULK1 by siRNA for 3 days. **F.**, Monitoring of apoptotic potential in 7 cell lines co-treated with PLX and CQ for 3 days. The level of cleaved caspase 3 was used to determine apoptosis.

MerTK is a member of the RTK family TAM (consisting of Tyro3, Axl and Mer), whose regulatory signaling is still poorly understood [24]. We analyzed the microarray data to compare signaling pathways co-activated with MerTK, and found that p85, the regulatory subunit of PI3K was elevated together with a moderate increase of mTOR, which was consistent with the observation of enhanced Akt phosphorylation (Supplementary Figure S2E). Therefore, we hypothesized that the mTOR/PI3K/Akt pathway might be affected upon BRAFi treatment. In addition, as MerTK is critical for macrophage engulfment, we examined the same microarray data for the signaling events related to endocytosis/phagocytosis. Interestingly, autophagy, a conserved lysosomal degradation machinery acting in response to nutrient deprivation and pathological stress [12], appeared to be enhanced. This was suggested by the simultaneous increase of two essential activators, ATG2B and ATG4A in two examined cell lines (Supplementary Figure S3A). Other factors important in the maintenance of autophagic activity, including ULK1 and ATG7, were also increased at the mRNA level (Supplementary Figure S3A). Consistently, PLX treatment promoted the cleavage of microtubule-associated protein 1A/1B-light chain 3 (LC3) and the degradation of p62, two key factors indicating activation of autophagy (Figure 3A). In fact, the mRNA level of MAP1LC3A was significantly increased in both A375p and A2058 cells exposed to PLX (Figure 3B). Functionally, LC3 puncta corresponding to LC3-labelled autophagosomes were considerably enriched in PLX-treated melanoma cells (Figures 3C, S3B), which was further demonstrated by the examination of labeled autophagosomes with electron microscopy (Supplementary Figure S3C). Pharmacological inhibition of autophagy by chloroquine (CQ) robustly abolished PLX-triggered MerTK overexpression (Figure 3D). Similarly, blocking the assembly of the autophagic initiating complex or the formation of autophagosomes by knocking down ULK1 or ATG7, respectively, vigorously suppressed both basal and induced MerTK expression (Figures 3E, S3D). As inhibition of autophagy at three different stages could suppress MerTK expression (Supplementary Figure S3E), these data illustrated a stringent dependence of MerTK upregulation on autophagy. A short treatment (24 h) of A375p cells pre-incubated with PLX (thus overexpressing MerTK) with CQ rapidly decreased MerTK levels (Supplementary Figure S3F), indicating that autophagy actively participates in the regulation of MerTK. In contrast, MerTK deficiency did not affect p62 degradation (Supplementary Figure S3G), demonstrating that the MerTK pathway is signaling downstream of autophagy. Simultaneous inhibition of mutant BRAF and autophagy efficiently induced caspase 3 cleavage in 7 melanoma cell lines (Figure 3F), which mimicked the phenotype of cells with loss of MerTK when exposed to BRAFi stress as previously observed (Figure 2C). These data demonstrated a pro-survival role of the autophagy/MerTK axis that desensitized melanoma cells to BRAFi-triggered apoptosis.

### Autophagy-dependent regulation of MerTK is mediated by BRAFi-triggered deactivation of mTORC1

**Figure 4:**
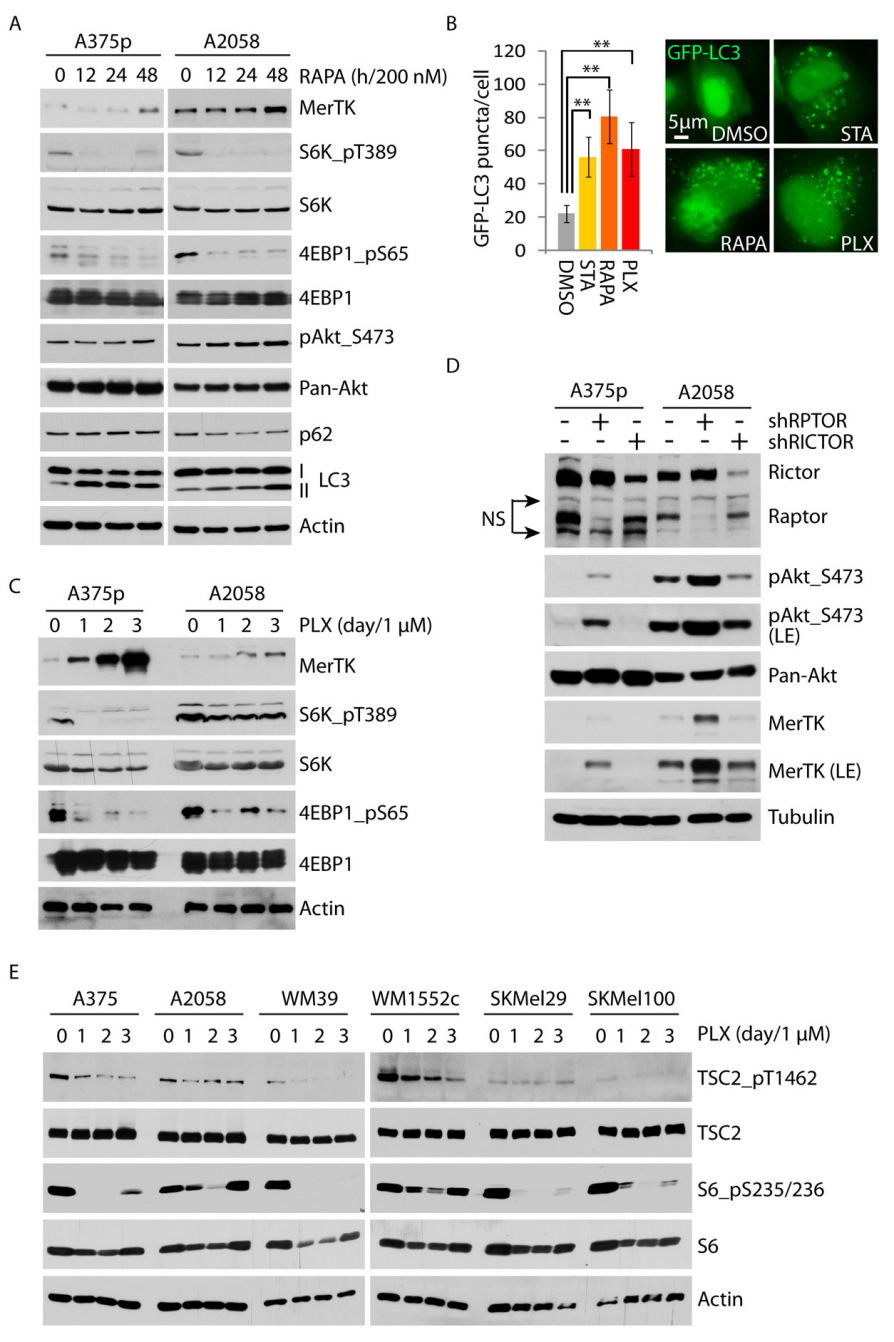
Vemurafenib upregulates MerTK through the mTORC1/autophagy signaling axis **A.**, A375p and A2058 cells were treated with 200 nM of rapamycin and the activities of mTORC1 (S6K_pT389/4EBP1_pS65) and autophagy (LC3 cleavage) were determined at the indicated time-points. **B.**, Quantification of GFP-LC3 puncta (labeled autophagosomes) in the presence of rapamycin (200 nM), vemurafenib (1 μM), or serum starvation (STA). GFP-LC3 was transiently expressed for 3 days and the treatment was administered for 2 days. **C.**, A375p and A2058 cells were treated with vemurafenib (1 μM) and the mTORC1 activity was determined by the phosphorylation level of its substrates S6K1 and 4EBP1 at the given time-points. **D.**, MerTK expression was determined in A375p and A2058 with knockdown of RPTOR and RICTOR (NS, non-specific). **E.**, Phosphorylation status of endogenous mTORC1 inhibitor TSC2 was examined in 6 melanoma cell lines treated with vemurafenib (1 μM) at indicated time-points.

One of the best characterized signalosomes that regulate autophagy is mTORC1. mTORC1 anchors and deactivates the autophagic complex on its surface *via* mTOR-directed phosphorylation of ULK1, the central kinase assembling the autophagy-initiating complex [25]. To examine whether autophagy-mediated MerTK activation could be mediated by mTORC1, we exposed melanoma cells to the pharmacological allosteric inhibitor rapamycin. In a time-course-dependent manner, MerTK expression increased within 48 hours in the presence of 200 nM of rapamycin and this was associated with a decrease in mTORC1-dependent phosphorylation of S6K1 (on Thr389) and 4EBP1 (on Ser65), as well as enhanced LC3 cleavage, indicating an enhancement of autophagy (Figure 4A). This is further demonstrated by increased functional assembly of autophagosomes in response to rapamycin (Figure 4B). Torin, an ATP-competitive mTOR inhibitor, could also moderately upregulate MerTK at low concentration (10 nM) in association with increased LC3 cleavage (Supplementary Figure S4A). Indeed, the phosphorylation level of both S6K and 4EBP1 was decreased in presence of PLX (Figure 4C), suggesting that inhibition of BRAF^V600E^ in melanoma cells resulted in downregulation of mTORC1 activity, which was also reported in an independent study recently [26]. In agreement with the outcome of rapamycin treatment, disruption of mTORC1 by genetic depletion of its key component Raptor, but not Rictor, activated MerTK expression (Figure 4D) and enhanced autophagosome formation (Supplementary Figure S4B). Taken together, the impact of mTORC1-regulated autophagy/MerTK resulted from inhibition of hyperactive mutant BRAF-driven MAPK signaling, indicating an interaction between these two signaling axes. In this context, mTORC1 activation could be regulated *via* two potential routes: ERK-mediated enhancement of Raptor, or ERK-mediated inhibition of tuberous Sclerosis Complex 2 (TSC2), a native intracellular inhibitor that restrains mTORC1 activation *in vivo*. In response to PLX treatment up to 3 days in 6 melanoma cell lines, ERK-regulated TSC2 phosphorylation is continuously reduced together with decreased phosphorylation of S6 on serine 235/236, indicating a functional release of TSC2 from ERK inhibition (Figure 4E), whereas the ERK-mediated Raptor phosphorylation is unchanged (data not shown). These data indicate that BRAFi triggers TSC2 activation that inhibits mTORC1, which leads to downstream signaling activation of autophagy/MerTK.

### MerTK is directly activated by epithelial-mesenchymal transition (EMT) regulator Zeb2

**Figure 5:**
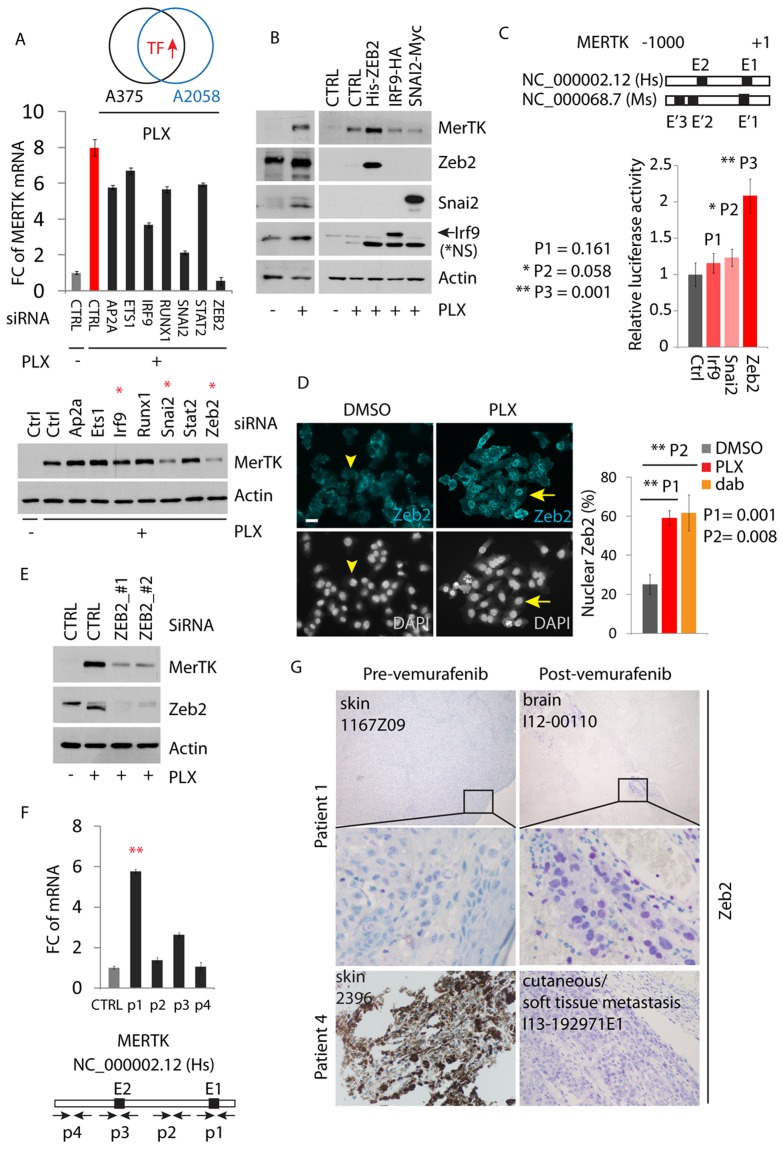
MerTK is a direct downstream target of Zeb2 **A.**, 7 transcription factors (TFs) that were upregulated in vemurafenib-treated A375p and A2058 melanoma cells were transiently knocked down and the level of MERTK mRNA was analyzed by qPCR (see also Figure S5C). The decreased MerTK expression at protein level was highlighted with red stars. B, Detection of MerTK expression in A375p cells expressing recombinant Zeb2, IRF9 and Snai2 after a 3 day course of vemurafenib therapy. **C.**, Luciferase reporter assay of transcriptional activity of Zeb2, IRF9 and Snai2 on MERTK promoter. Individual E-boxes located within MERTK promoter region (1kb) were highlighted in black squares and the corresponding sequences were listed. P value was calculated with student *t*-test. **D.**, Immunostaining of endogenous Zeb2 in A375p cells treated with vemurafenib. Nuclear translocation of Zeb2 was counted in A375p cells treated with either vemurafenib or dabrafenib (1 μM). **E.**, Detection of MerTK expression in A375p cells expressing two different pairs of ZEB2 siRNA in the presence of vemurafenib. **F.**, Chromatin was immunoprecipitated with the indicated antibodies [immunoglobulin G (IgG) as negative control] in A375p cells expressing recombinant Zeb2. Corresponding amplified DNA fragments were indicated in the promoter region of MERTK (the primers used are listed in supplementary Table S1). **G.**, Immunohistochemical staining of Zeb2 in two paired human melanomas before and after vemurafenib therapy. Purple staining indicates Zeb2. Note: the dark brown is not from the staining but due to the natural pigment melanin.

Although the precise molecular mechanism was still unknown, several studies indicated that RTK activation in response to BRAFi in melanoma was possibly a transcriptional event [9]. This was confirmed in our hands since downregulation of MerTK by inhibition of autophagy was due to decreased MERTK transcription (Supplementary Figure S5A) but not to a change of protein stability (Supplementary Figure S5B) as reported during the regulation of retinal phagocytosis [27]. To explore the molecular details how MERTK was regulated, we analyzed the transcription factors that were co-upregulated in two melanoma cell lines (A375p and A2058) treated with PLX. Based on the mRNA fold-change, the top 7 (out of 22) candidates were selected for evaluation. These 7 genes were transiently knocked down and the MERTK mRNA was measured by qPCR. When the mRNA of these 7 genes was targeted by individual siRNAs (Supplementary Figure S5C), the MERTK level was significantly affected by SNAI2 and ZEB2 depletion, but only mildly reduced with IRF9 knockdown compared to control (Figure 5A, red bar). This influence of the depletion of SNAI2 and ZEB2, rather than IRF9, was also reflected at the protein level (Figure 5A, red stars). However, unlike Zeb2, in presence of PLX, overexpression of Irf9 and Snai2 did not enhance MerTK expression (Figure 5B). Similarly, expression of Irf9 failed to increase MERTK-promoter-driven luciferase activity, which was significantly promoted with Zeb2 expression but only slightly with Snai2 expression (Figure 5C), implying that Zeb2 may be a major regulator of the MERTK promoter. Indeed, PLX-treatment induced Zeb2 accumulation in the nucleus (Figure 5D). Transient downregulation of Zeb2 with either of two distinct pairs of siRNAs effectively blocked BRAFi-induced MerTK expression (Figure 5E). The promoter region of MERTK contains a conserved E-box (E1) close to the transcription start that represents a potential binding site for E-box-binding proteins such as Zeb2. Within 1kb upstream of transcription start, there are also other E-boxes on both human and mouse promoters of MERTK. To investigate if Zeb2 can bind to these core enhancers, we performed a chromatin immunoprecipitation (ChIP) assay using overexpressed human Zeb2 in A375p melanoma cells. In the presence of PLX, Zeb2 preferentially binds to the proximal promoter region of human MERTK harbouring E-box 1 (E1), although mild binding to the E-box 2 (E2) could also be observed to a certain extent (Figure 5F). These results indicate that Zeb2 is capable of transcriptionally upregulating MERTK *via* direct binding to its promoter, whose activation controls MerTK expression in melanoma cells upon inhibition of mutant BRAF. In agreement with the *in vitro* studies, in the four paired human melanomas where MerTK is overexpressed post-vemurafenib therapy (Figure 1D), nuclear accumulation of Zeb2 is broadly present in resistant tumors (Figure 5G). However, the brain metastases from patient 8 showed equally high levels of Zeb2 before treatment compared with lymph node metastases after treatment, while MerTK was only mildly detectable (Supplementary Figure S5E). In patient 7, although MerTK was indistinguishably overexpressed in both pre- and post-vemurafenib treated tumors, the Zeb2 level was significantly higher in relapsed brain metastases after treatment (Supplementary Figure S5F). These observations suggest that except for Zeb2, MerTK is possibly regulated by other transcriptional events; vice versa, Zeb2 may transcriptionally regulate other downstream targets to confer resistance.

### Inhibition of autophagy/MerTK overcomes BRAFi-induced resistance in a xenograft model

**Figure 6:**
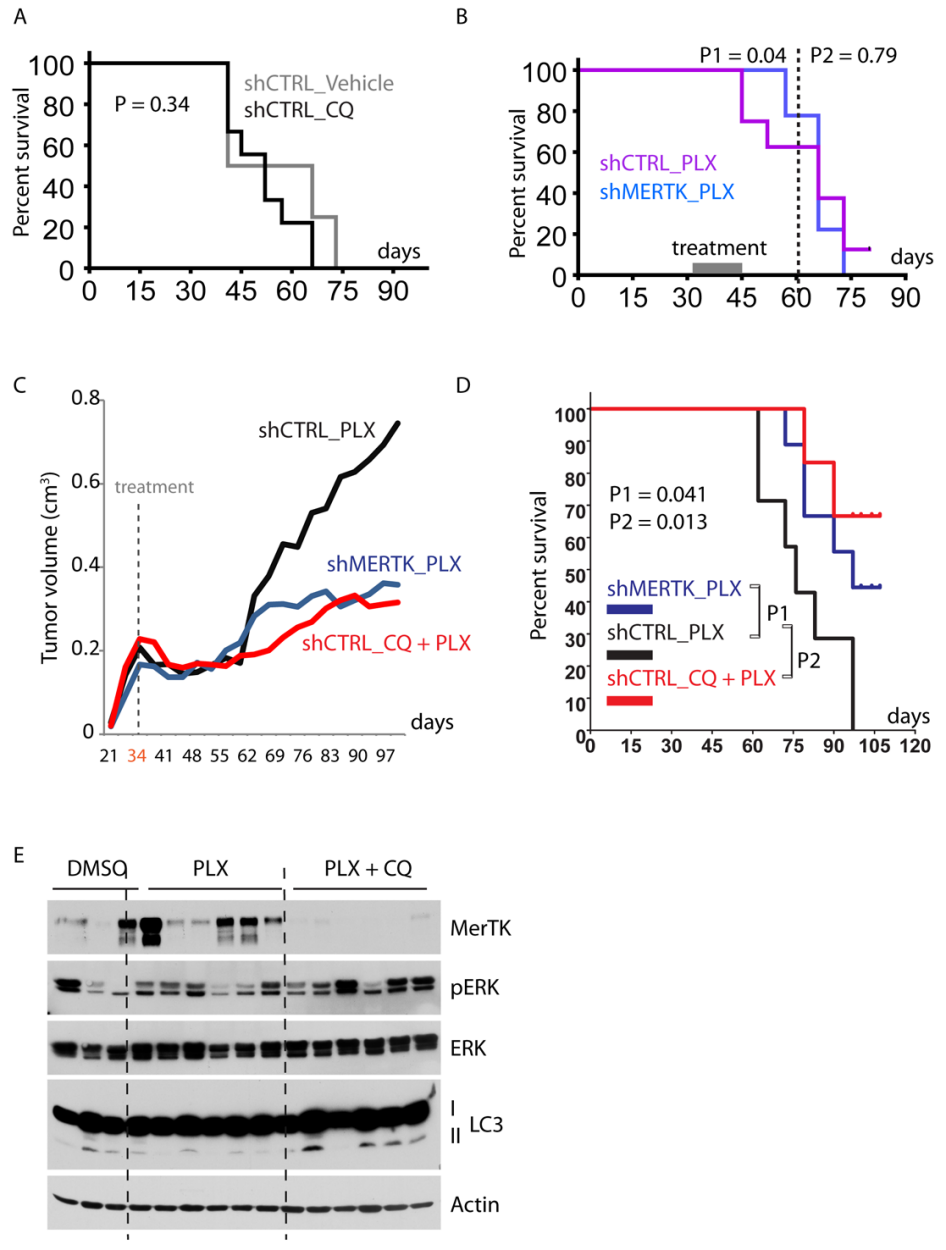
Combined inhibition of mutant BRAF and autophagy/MerTK overcomes resistance to vemurafenib therapy **A.**, Survival analysis of mice inoculated with A375p_shCTRL cells and treated with vehicle or CQ (twice a week). **B.**, Survival analysis of nude mice inoculated with A375p_shCTRL or A375p_shMERTK cells and treated with PLX for 14 days (acute treatment). Loss of MerTK improved animal survival before day 60 (*P*1 = 0.04) but this effect was lost on day 80 (*P*2 = 0.79). **C.**, Growth curve of xenograft tumours in mice treated with PLX, PLX+CQ and PLX+shMerTK. The treatment started on day 34 and stopped on day 97. The tumour size was measured twice a week. Each group contained 9∼10 mice. **D.**, Kaplan-Meier survival analysis of nude mice injected with A375p_shCTRL or A375p_shMERTK and treated with PLX or Combo (CQ + PLX). Daily treatment started on day 34 (when the tumour volume was between 0.15 ∼ 0.3 cm^3^) and stopped on day 97. The mice were necropsied when the tumor volume reached ∼ 1 cm^3^. Each group contained 8∼10 mice. **E.**, Analysis of MerTK expression in the xenograft tumours treated with PLX or combo (PLX+CQ). See also Figure 2E.

Due to tumour relapse *in vivo* (Figure 2E), mice injected with MerTK-depleted A375p melanoma cells did not have longer survival (Supplementary Figure S6A), and isolated CQ-treatment did not induce tumour regression (Figure 6A). In contrast, PLX-treatment (starting from day 34) potently inhibited tumor growth during the exponential growth phase (day 34∼60), although the tumours started to re-grow when the mice were still under daily PLX treatment (day 60∼97) (Supplementary Figure S6B). The re-growth phase likely reflected *in vivo* resistance to vemurafenib that subsequently resulted in regained tumor growth. Thus, the tumor load of PLX-treated mice was similar to that of mice treated with the vehicle or CQ alone (Supplementary Figure S6C). However, combined treatment with CQ and PLX showed a significantly improved anti-tumor activity over single treatment with PLX, even during the acute treatment for 14 days (Supplementary Figure S6D). Under the same condition, the mice bearing MerTK-depleted tumours responded to BRAFi better than control mice bearing MerTK-expressing tumours after the treatment was stopped for 2 weeks (P1, day 60), but the response was lost after 4 weeks (P2, day 75) (Figure 6B), indicating that MerTK depletion at early time point potentially sensitized tumour response to vemurafenib *in vivo*. In order to evaluate long-term effect of MerTK depletion combined with vemurafenib treatment, we treated the mice bearing MerTK-deficient tumours with PLX for 8 weeks once daily. Long-term treatment with PLX and CQ abolished tumour regrowth compared with single treatment with PLX, which was pheno-copied by single PLX treatment combined with MerTK depletion (Figures 6C, S6B). The survival rate of mice bearing deficient MerTK treated with PLX was 100% at day 60, and ∼50% at day 97 in the long-term treatment, which was similar to the combinatorial treatment with CQ and PLX (∼70%) in mice with intact MerTK (Figure 6D). In contrast, although prolonged monotherapy with PLX improved mouse survival (∼55% on day 75) compared with short-term treatment (∼15% on day 75, Figure 6B), no mouse survived beyond day 97. These data demonstrated that MerTK overexpression can reduce the response of tumors to vemurafenib by protecting melanoma cells from environmental stress-induced apoptosis. In fact, PLX treatment vigorously induced MerTK expression in xenograft tumours, which was robustly inhibited upon inhibition of autophagy with CQ (Figure 6E). Thus, co-targeting BRAF^V600E^ and MerTK may substantially reduce tumour burden in mice, which pheno-copied the outcome of dual-inhibition of BRAF^V600E^ and autophagy.

## DISCUSSION

The major challenge in clinical cancer therapy is drug-induced resistance that leads to unresponsiveness of tumor cells to a pharmacologically stressed environment [28, 29]. Hyperactivation of protective cellular signaling pathways, such as RTKs, PI3K/Akt, MAPK and autophagy, is almost universally observed in clinical cancer therapy. These protective signaling pathways are responsible for the occurrence of cancer resistance and relapse. The mechanisms of resistance have remained somewhat elusive [30]. Although recent studies revealed that reactivation of RTKs was one of the major drivers of resistance against BRAF inhibitors, little is known how this resistance is mechanistically modulated. In addition, although autophagy is required for cell survival [13, 31] and clinical studies confirm its global activation in tumors [32], isolated pharmacological suppression of autophagy did not meet success in clinical trials. Similarly, targeting of mTORC1 by rapalogs in cancers also induced rapid clinical resistance, whose mechanisms are currently under extensive investigation [33].

Our data highlight a novel mechanism of mTORC1/autophagy/MerTK-mediated resistance to BRAFi in melanoma harbouring BRAF^V600E^ mutation (Supplementary Figure S6E). Unlike BRAFi, MEKi such as trametinib did not affect MerTK in tested cell lines with acquired resistance to trametinib (Supplementary Figure S1J). Importantly, trametinib did not block MerTK expression in BRAFi-resistant A375R cells, or in A375R with acquired resistance to both BRAFi and MEKi (A375R2) (Supplementary Figure S1J). Interestingly, this observation in established cell lines could be confirmed in biopsies from BRAFi/MEKi resistant human melanomas (Figure 1E), although more clinical melanomas with acquired resistance to combined BRAFi+MEKi should be examined. Nevertheless, our data not only demonstrates a stringent control of MerTK expression as a consequence of BRAF inhibition, but also hints at a potential role of MerTK as a mediator of resistance against combination therapy. Being the core machinery of host defense and survival, phagocytosis requires MerTK activation [34, 35] and cross-talks to autophagy [36, 37], another metabolic and homeostatic regulating machinery adopted by cancer cells to improve their fitness [38]. In line with recent studies [20, 39], MerTK is emerging as an important regulator in tumorigenesis and metastasis, possibly playing a unique role at the crossroads between tumour cells and the host immune system.

In presence of BRAFi, Zeb2 is significantly activated in melanoma cells. Zeb2 is a member of the δEF-1 protein family containing a homeodomain flanked by highly conserved zinc finger clusters at both termini [40]. During embryogenesis, genetic depletion of Zeb2 leads to impaired mouse embryo development from E8.5, associated with early arrest of cranial neural crest cell migration and absence of neural crest cells [41]. This is a typical phenotype resulting from a defective function of EMT regulators such as Twist, Snai1/2, Zeb1/2 and E47 [42]. Mechanistically, these EMT-driving transcription factors are activated in response to a variety of stimuli and subsequently lead to cell reprogramming [43] through mediating signaling crosstalk among different oncogenic pathways such as TGFβ [44, 45] and Notch [46], and significantly contribute to drug resistance in clinical cancer therapy [47]. However, in melanomas harbouring mutant BRAF, Zeb2 is dynamically switched on/off and inversely associated with MAPK activation [48]. In agreement with previous observations, our data showed reactivation of Zeb2 in response to BRAFi and consequent overexpression of MerTK. Thus, in the context of mutant BRAF inhibition in melanoma, the activation of Zeb2/MerTK signaling plays an important role in mediating cancer cell survival and resistance. Interestingly, the Zeb2-associated overexpression of MerTK was not observed in two paired melanomas (Supplementary Figures S5E and S5F). Although further quantitative analysis is needed, it seems that when high levels of MerTK are present before treatment starts, the tumor cells may take advantage of other mechanisms to survive BRAF or combined BRAF/MEK blockade.

Despite various investigated underlying mechanisms, including reactivation of the PI3K/Akt through a feedback loop, the parallel activation of MAPK axis, and Myc-driven oncogenesis, our data reveal that autophagy-regulated MerTK is crucially involved in promoting melanoma cell survival to resist BRAF^V600E^ inhibition. This was confirmed by functional inhibition of the mTORC1 activity, as well as blockage of autophagy at different stages. Although autophagy has been considered a druggable target in cancer therapy, in agreement with previous *in vivo* studies [49], we showed that CQ treatment had no overall impact on A375p-xenograft tumour regression in mouse model and current clinical results are also disappointing. To a certain extent this is consistent with our observations that MerTK depletion (partly mimicking the consequence of autophagy inhibition in our model) delayed tumour formation but was not sufficient to block tumorigenicity, although other mechanisms could also be involved (Supplementary Figure S1I, patient 5). Nevertheless, co-inhibition of BRAF^V600E^ with MerTK or autophagy remarkably improved progression-free survival. Interestingly, a recent study demonstrated that inhibition of autophagy may overcome BRAFi-induced resistance [50], and a combinatorial inhibition of mTOR and autophagy in a clinical phase I trial in patients with advanced melanoma patients has shown improvement of anti-tumor activity [51]. Therefore, we propose that simultaneous targeting of BRAF^V600E^ and autophagy/MerTK signaling axes is a rational strategy for overcoming resistance to BRAF inhibitors in melanoma.

## MATERIALS AND METHODS

### *In vivo* studies of drug inhibition

0A375 melanoma cells and its derivatives were subcutaneously injected into nude mice (Harlan France). PLX-4720 (stocked in DMSO and diluted in PBS containing 1% methylcellulose and 0.2% Tween 80) was administered orally at 45 mg/kg body weight daily [52]. CQ (dissolved in 0.9% NaCl) was injected twice a week at 50 mg/kg body weight [49]. The compounds were freshly prepared before treatment. The treatments started when flank tumours reached ∼150 mm^3^. In the short-term experiment, the tumour-bearing mice were treated daily from day 31 for 14 days (day 31∼45), following drug-free maintenance under standard conditions for additional 5 weeks (day 46∼80). In the experiment investigating long-term therapy, the daily treatment with PLX started on day 34 and ended on day 97. CQ was injected twice a week. Each group contained 8∼10 mice and the tumours were resected when the volume reached ∼1 cm^3^ (in accordance to Swiss Animal Protection Ordinance). The survival data and P value were presented with GraphPad Prism. See also supplemental methods.

## SUPPLEMENTARY MATERIALS FIGURES AND TABLES


