# Supplementary material for: mTORC1/autophagy-regulated MerTK in mutant BRAFV600 melanoma with acquired resistance to BRAF inhibition
Source: Oncotarget. 2017 May 25;8(41):69204–18. doi: 10.18632/oncotarget.18213 (PMC5642473; doi:10.18632/oncotarget.18213)
Supplement: Supplementary file 1 [file oncotarget-08-69204-s001.pdf]

mTORC1/autophagy-regulated MerTK in mutant BRAFV600 melanoma with acquired resistance to BRAF inhibition

Supplementary Information

METHODS:

Human Melanoma Cell Lines. A375p, Mel-1300, GR4 and DETT-Mel were described previously [1]; A2058, SKMel2, SKMel23, MalMe, MalMe-3M, HBL, C32 and NA8 were provided by Matthias Wymann [2]; LAU-T672E, LAU-Me246.M1, LAU-T392E, and LAU-T387B were established and characterized at the Ludwig Institute for Cancer Research (Lausanne) [3]; SKMel19, SKMel29 and SKMel100 were from the Memorial Sloan-Kettering Cancer Center [4]; WM35, WM39, WM278, WM793b, and WM1552c were purchased from the Wistar Institute. The mutation status of BRAF is as following:

wild-type BRAF: Mel-1300, GR4, DETT-Mel, SKMel2, SKMel23, SKMel19, NA8, HBL, MalMe;

mutant BRAF: A375p (V600E), A2058 (V600E), C32 (V600E), MalMe-3M (V600E), LAU-T672E (V600E), LAU-Me246.M1 (V600E), LAU-T392E (V600E), LAU-T387B (V600E), SKMel29 (V600E), SKMel100 (V600E), WM35 (V600E), WM39 (V600E), WM278 (V600E), WM793b (V600E), WM1552c (V600E).

Establishment of the Resistant Cell Lines. A375p, A2058 and SKMel100 were cultured in DMEM supplied with 1 µM of PLX-4720 or trametinib 0.1 µM for two months. Double resistance to dabrafenib+trametinib was achieved (for A375p) by incubation with 1 µM

dabrafenib and 0.1 μM of trametinib for two months and the resulting resistant cell line was named as A375R2.

Human Melanomas. Human melanomas were obtained from the University Hospital of Zurich (Biobank, Department of Dermatology, Zurich, Switzerland), the University Hospital of Basel (Department of Biomedicine and Institute of Pathology, Basel, Switzerland) and the Papa Giovanni XXIII Hospital (Unit of Clinical and Translational Research, Bergamo, Italy). The use of completely anonymized human tumour samples for retrospective studies was sanctioned by the Ethics Commission of Papa Giovanni XXIII Hospital (approval No. prot./CE n. 186, Bergamo, Italy), Zurich (approval No. 647, Switzerland) and Basel (EKBB, Switzerland).

Reagents. PLX-4720 and trametinib, Torin, Chloroquine, rapamycin, BEZ235 and U0126 were bought from Selleck Chemicals, Sigma-Aldrich, MerckMillipore, Novartis and Cell Signaling Biotechnology, respectively. shRNA targeting human MERTK was purchased from Open Biosystems, and shRNA targeting human ATG7 was a gift from Masashi Narita. shRNAs targeting RPTOR and RICTOR [5] were from Addgene (Table S1). siULK1 was from Cell Signaling Biotechnology and other siRNAs were synthesized by Microsynth (Switzlerand). pcDNA-His-Zeb2 was kindly offered by Janet Mertz (University of Wisconsin-Madison), and IRF9-HA and Snai2-myc were from Addgene. The antibodies against MerTK (FMI and Abcam), Raptor and Rictor (Bethyl Laboratories), pan-Akt, Akt_pS473, S6K, S6K_pT389, S6, S6_pS235/236, 4EBP1, 4EBP1_pS65, cleaved caspase 3, pMLC2, ULK1, Atg7, LC3, p62, ERK, and pERK (Cell Signaling Biotechnology), fibronectin (BD Biosciences), Zeb2 (Sigma) and actin (Santa Cruz Biotechnology) were applied according to the suppliers’ instructions. The BioCoat Matrigel invasion chambers were from BD Biosciences.

Xenograft Tumour Lysis. Resected tumours were shortly rinsed with PBS and immediately flash frozen in liquid nitrogen and stored at -80˚C. Tumour blocks were directly incubated with 1X RIPA buffer containing 25 mM Tris-HCl pH 7.4, 150 mM NaCl, 1% NP-40, 1 mM EDTA, 5% glycerol and supplemented with protease inhibitor cocktail (Roche). Lysis was performed with mechanical tissue disruptor on ice.

Short-term Proliferation Assay. Cultured melanoma cells were seeded into 6-well plates at 1-3 x 10^4 per well in the presence/absence of individual drugs for 72 ~ 96-h. The medium was changed daily and cell number/viability was measured with Vi-Cell XR (Beckman Coulter). Samples were prepared in triplicates.

Long-term Proliferation Assay. A375p, A2058 and SKMel100 cells were seeded into 6-well plates at 500 cells per well in the presence/absence of PLX (1 or 2 μM) for 12 days or 24 days. The treatment was daily. The colonies were fixed with PFA and stained with crystal violet for 30 min, followed by 3 times of rinsing with PBS, air-drying and photography of the entire plates. The size and the number of colonies were measured with ImageJ. Samples were prepared in triplicates.

Apoptosis Assay. Melanoma cells were seeded into 6-well plates in the presence/absence of compounds for 24-72 h. Apoptosis was measured by Annexin V staining (BD Bioscience) and analyzed by FACSCalibur. The results were from three independent experiments and presented as mean ± standard deviation.

Chromatin Immunoprecipitation (ChIP)

ChIP assay was principally performed with the same protocol as described [6]. In summary, A375p melanoma cells were harvested and treated with 1% formaldehyde for 10 minutes and subjected to lysis and sonication. Pre-cleared with protein G Sepharose (GE Healthcare), the lysates were incubated with protein G conjugated with individual antibodies overnight. The associated DNA was extracted and purified for qPCR analysis.

Transmission Electron Microscopy (TEM). A375p cells were grown on Thermanox (Nalge Nunc International) coverslips and treated with Vemurafenib (1 µM) for 3 days, following rapid rinse with PBS warmed at incubation temperature and fixed with 2% PFA and 2.5% glutaraldehyde in 0.1 M PBS pH 7.4. Cells on coverslips were stained with 1% osmium and 1.5% potassium ferrocyanide followed by 1% osmium. After rinsing, cells were stained with 1% uranyl acetate. Following dehydration, cells were flat-embedded in Embed812 (EMS). Coverslips were removed by plunging in liquid nitrogen and thin sections (50 nm) were collected on formvar-coated slot copper grids (EMS). Images were taken with a Veleta camera (Olympus) on a CM10 transmission electron microscopy [7]. 20 images per treatment have been taken and autophagosomes have been counted manually using FIJI.

Transcriptome Analysis and QPCR. Microarray analysis was performed using the Affymetrix "Human Gene 1.0 ST arrays" following the instructions provided by the manufacturer (referring to the GEO submission for details). A375p and A2058 cells were treated with PLX-4720 (1 µM) for 3 days by applying fresh solution each day. An equal volume of DMSO was used as control. Each treatment was performed in triplicate. QPCR was performed with the StepOnePlus Real-Time PCR System (Applied Biosystems). Primer design was based on the "Primer databank" of Harvard University and validated. Microarray data was analyzed using R (v 3.0.1) and

Bioconductor packages. In brief, raw data was normalized using RMA as implemented in the affy package [8] and statistically significant differentially expressed genes were determined using the linear modelling of the limma package [9]. Indicated p-values were corrected for multiple testing using the Benjamini-Hochberg algorithm. Raw and normalized microarray data is available in the GEO dataset GSE52882.

Assays of Xenograft Tumour Formation and Lung Metastasis. Animal maintenance and experimental procedures complied with the Swiss and Italian Animal Protection Ordinances. A375p and its derivatives (10^6 cells) were subcutaneously injected into nude mice (Charles River Laboratories). The flank tumours were measured weekly, flash-frozen and lysed in RIPA buffer containing protease inhibitor cocktail (Roche). For metastasis assays, 5×10^5 A375 cells (in PBS) were injected into the tail vein of CB.17 SCID mice. The lung macrometastases were dissected microscopically 49 days post-injection, flash-frozen, and homogenized in lysis buffer (125 mM Tris-HCl, 2.5% SDS, pH 6.8).

Statistical Methods

Quantitative results are analyzed from at least three independent experiments. Quantitative data were prepared using the Student's t test. All statistical tests were two-sided, and error bars in the graphs represent standard deviations. Graphics were analyzed using the GraphPad Prism program.

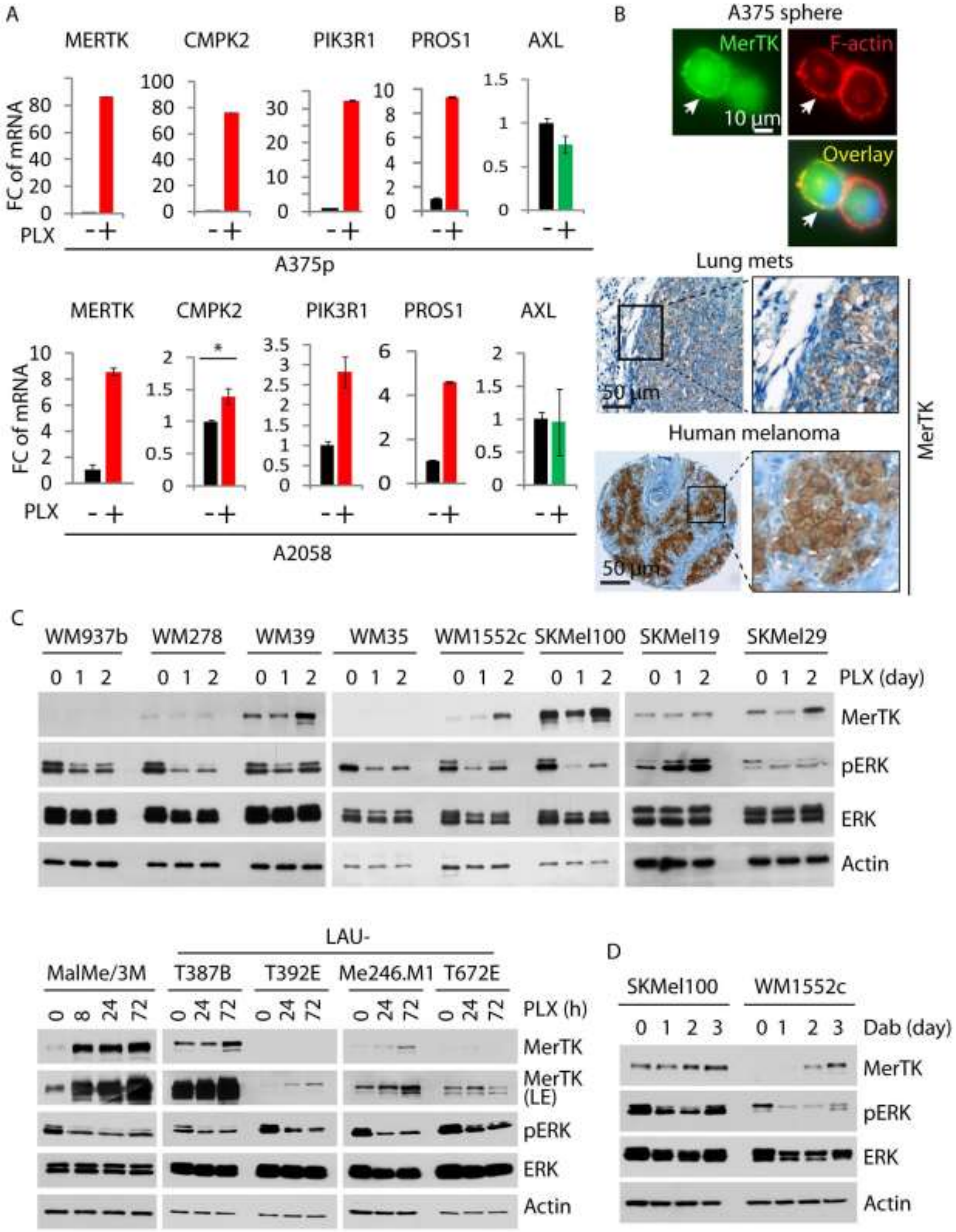
A
MERTK
CMPK2
PIK3R1
PROS1
AXL
FC of mRNA
PLX
A375p
MERTK
CMPK2
PIK3R1
PROS1
AXL
FC of mRNA
PLX
A2058
B
A375 sphere
MerTK
F-actin
10 µm
Overlay
Lung mets
50 µm
Human melanoma
50 µm
MerTK
C
WM937b
WM278
WM39
WM35
WM1552c
SKMel100
SKMel19
SKMel29
PLX (day)
MerTK
pERK
ERK
Actin
LAU-
MalMe/3M
T387B
T392E
Me246.M1
T672E
PLX (h)
MerTK
MerTK (LE)
pERK
ERK
Actin
D
SKMel100
WM1552c
Dab (day)
MerTK
pERK
ERK
Actin

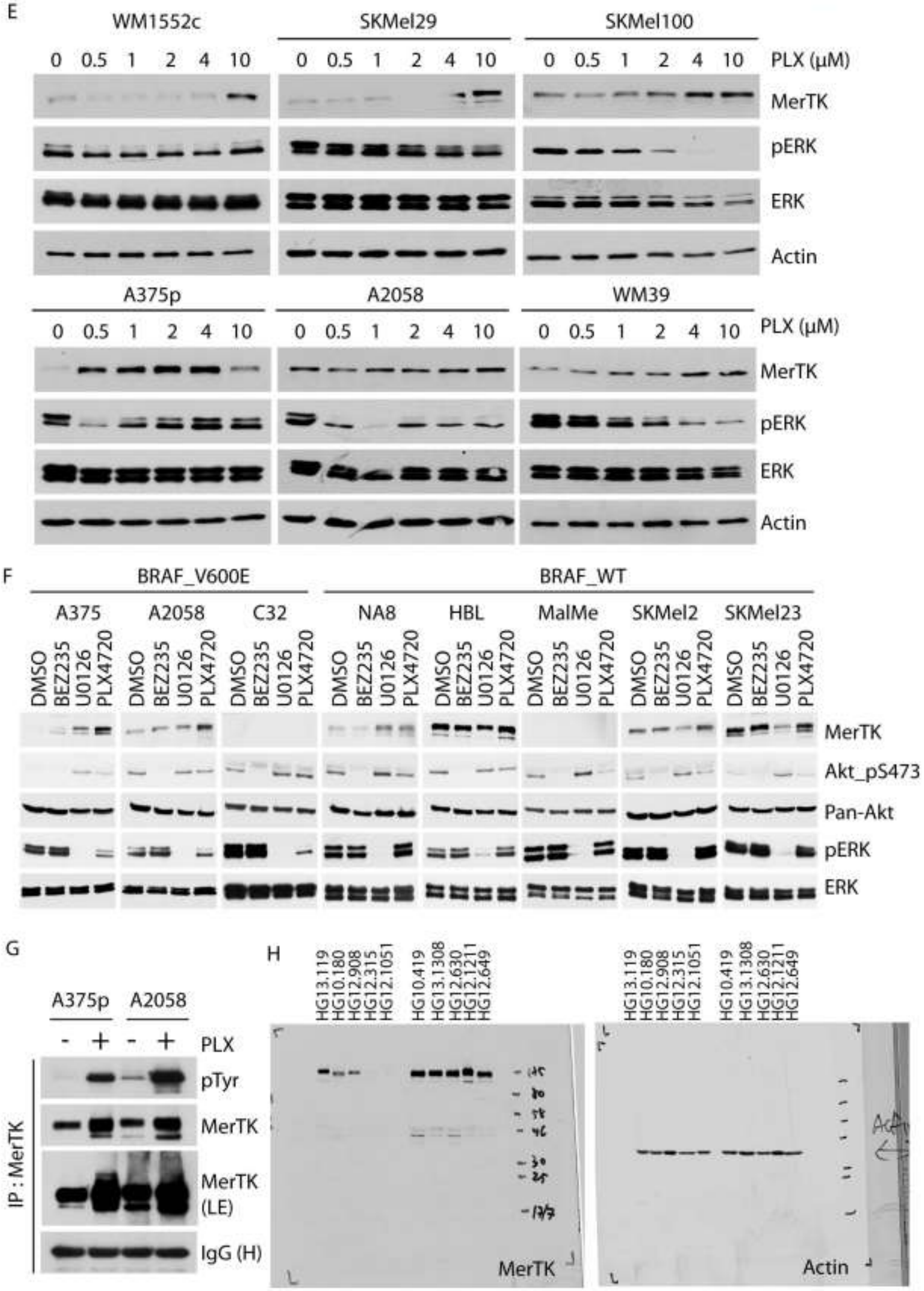
E
WM1552c
SKMel29
SKMel100
0 0.5 1 2 4 10
PLX (μM)
MerTK
pERK
ERK
Actin
A375p
A2058
WM39
F
BRAF_V600E
BRAF_WT
A375
A2058
C32
NA8
HBL
MalMe
SKMel2
SKMel23
DMSO
BEZ235
U0126
PLX4720
MerTK
Akt_pS473
Pan-Akt
pERK
ERK
G
A375p
A2058
- + - +
PLX
pTyr
MerTK
MerTK (LE)
IgG (H)
IP : MerTK
H
HG13.119
HG10.180
HG12.908
HG12.315
HG12.1051
HG10.419
HG13.1308
HG12.630
HG12.1211
HG12.649
MerTK
Actin

I

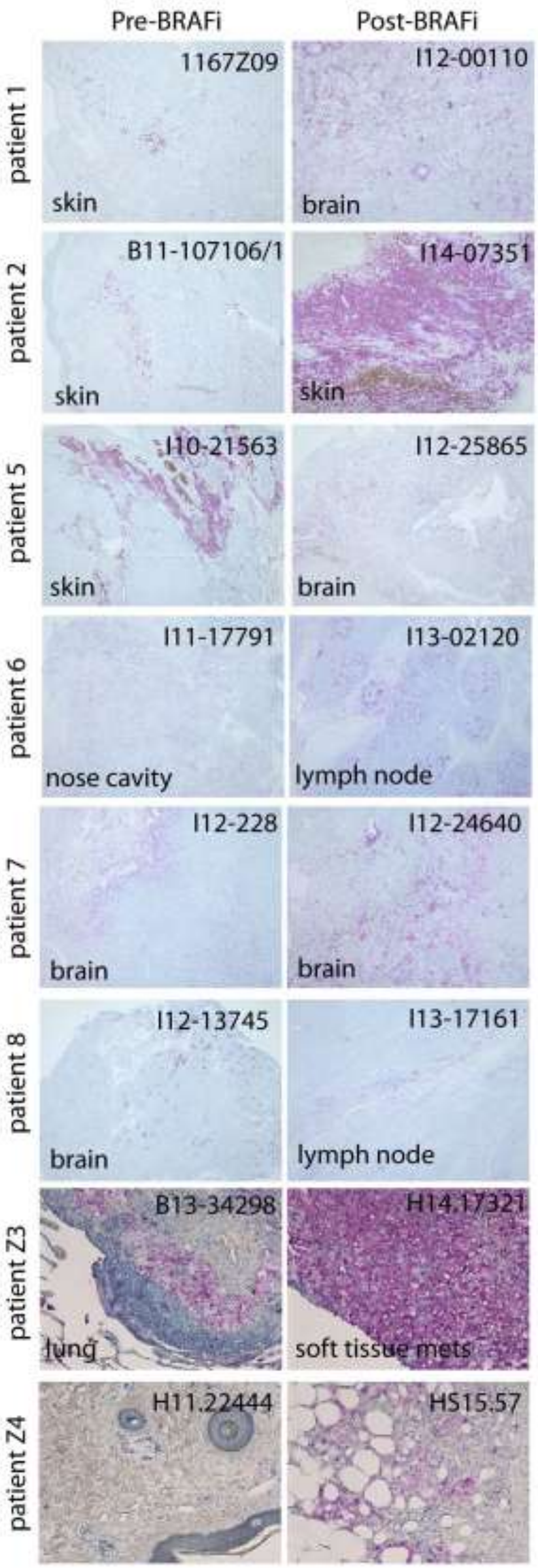
Pre-BRAFi
Post-BRAFi
patient 1
1167Z09
skin
I12-00110
brain
patient 2
B11-107106/1
skin
I14-07351
skin
patient 5
I10-21563
skin
I12-25865
brain
patient 6
I11-17791
nose cavity
I13-02120
lymph node
patient 7
I12-228
brain
I12-24640
brain
patient 8
I12-13745
brain
I13-17161
lymph node
patient Z3
B13-34298
lung
H14.17321
soft tissue mets
patient Z4
H11.22444
HS15.57

J

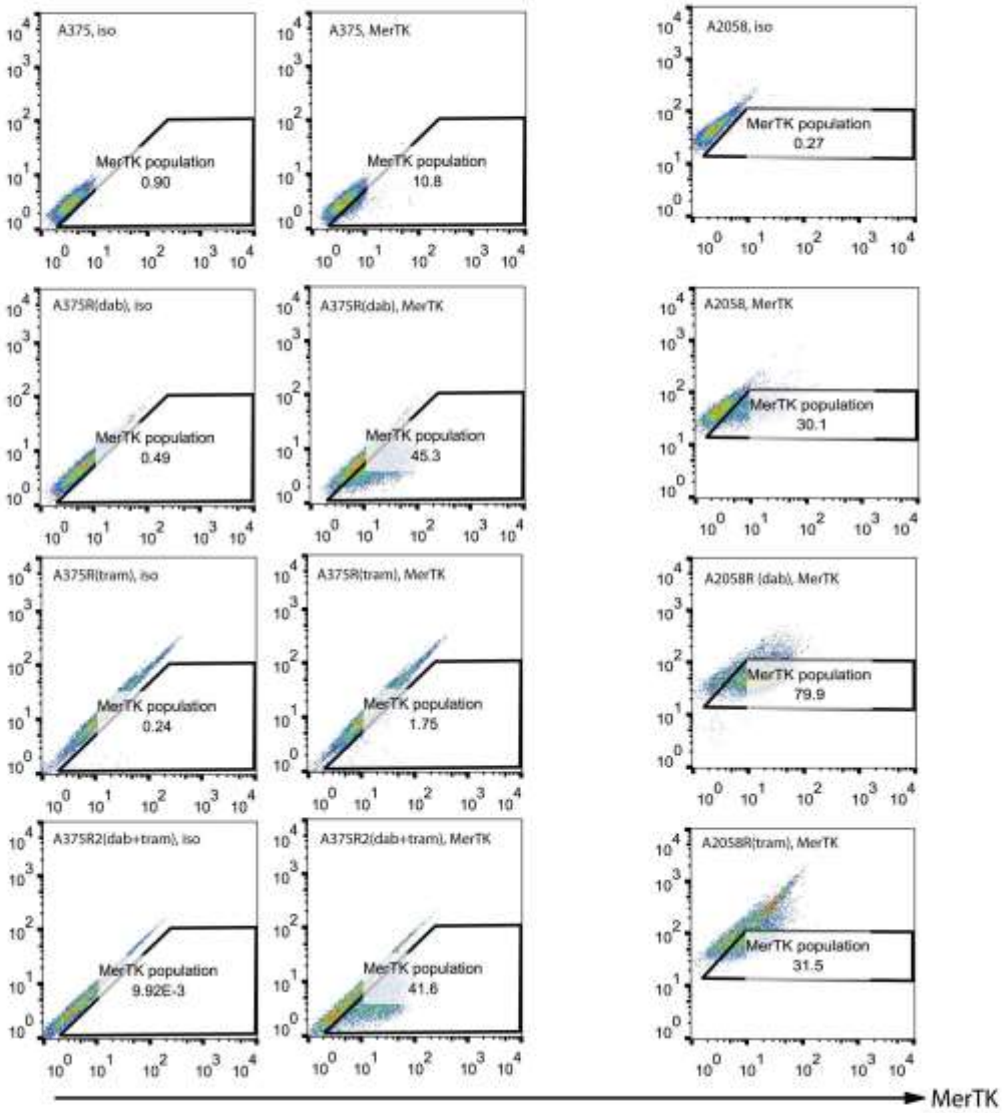
A375, iso
MerTK population
0.90
A375, MerTK
MerTK population
10.8
A2058, iso
MerTK population
0.27
A375R(dab), iso
MerTK population
0.49
A375R(dab), MerTK
MerTK population
45.3
A2058, MerTK
MerTK population
30.1
A375R(tram), iso
MerTK population
0.24
A375R(tram), MerTK
MerTK population
1.75
A2058R (dab), MerTK
MerTK population
79.9
A375R2(dab+tram), iso
MerTK population
9.92E-3
A375R2(dab+tram), MerTK
MerTK population
41.6
A2058R(tram), MerTK
MerTK population
31.5
MerTK

K

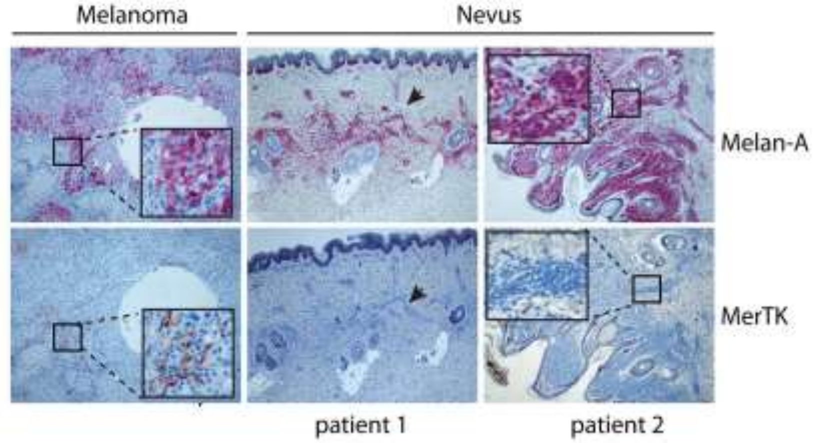
Melanoma
Nevus
Melan-A
MerTK
patient 1
patient 2

Supplementary Figure 1 A, Quantitative PCR analysis of the mRNA level of the indicated targets in PLX-4720-treated A375p and A2058 melanoma cells (the same RNA samples submitted to microarray analysis). **B**, Immunostaining of MerTK expression and localization in A375p-derived spheres (cultured in low-attachment dish for 3 days; white arrow pointing to the plasma membrane that showed MerTK signal, which was absent on the adjacent cell membrane), A375p-derived lung metastases in mice and human melanomas. **C**, Analysis of MerTK expression in 13 $BRAF^{V600E}$ melanoma cell lines treated with PLX-4720 (1 μM) at the indicated time-points. **D**, Examination of MerTK expression in SKMel100 and WM1552c melanoma cells treated with dabrafenib (1 μM) at the indicated time-points. **E,** Examination of the dose-response of MerTK expression to vemurafenib in 6 $BRAF^{V600E}$ melanoma cell lines (treated for 2 days). **F**, Investigation of MerTK expression in 5 $BRAF^{WT}$ melanoma cell lines treated with BEZ235 (2 μM), U0126 (50 μM) and PLX-4720 (1 μM), respectively. 3 melanoma cell lines harboring $BRAF^{V600E}$ were used as control. **G,** Endogenous MerTK was immunoprecipitated from A375p and A2058 cells treated with PLX-4720 (1 μM) for 6 days. The MerTK activity was determined by the level of its tyrosine phosphorylation. LE: long exposure. **H,** Comparison of MerTK level in 10 human melanomas (from 10 individual patients) before and after vemurafenib therapy. **I,** Immunohistochemical staining of MerTK in 8 pairs of paired human melanomas pre-/post-BRAFi therapy. Purple staining represents MerTK. **J,** FACS analysis of MerTK expression in A375p with acquired resistance to dabrafenib (A375R(dab)), trametinib (A375R(tram)) and dabrafenib+trametinib (A375R2(dab+tram)), as well as in A2058 with acquired resistance to dabrafenib (A2058R(dab)) and trametinib (A2058R(tram)). Isotype IgG was used as staining control. The number represents the percentage of MerTK+ cells. **K,** Examination of MerTK

expression in two samples of human melanocytic nevi. One melanoma was used as control. Purple staining and brown staining represent Melan-A and MerTK, respectively.

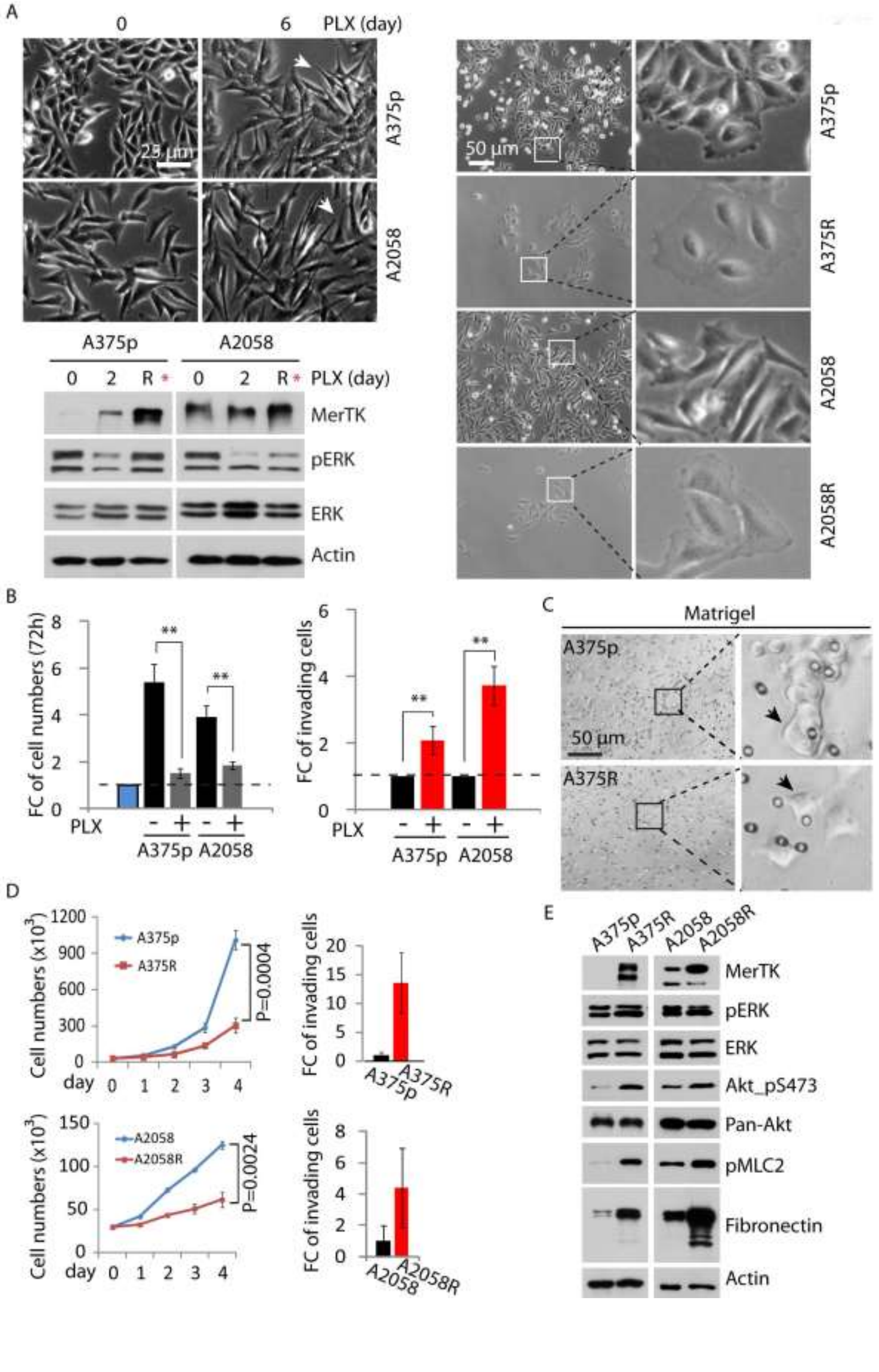

A
0
6
PLX (day)
25 µm
A375p
A2058
50 µm
A375p
A375R
A2058
A2058R
A375p
A2058
0 2 R 0 2 R PLX (day)
MerTK
pERK
ERK
Actin
B
FC of cell numbers (72h)
FC of invading cells
PLX
A375p A2058
C
Matrigel
A375p
A375R
50 µm
D
Cell numbers (x10³)
A375p
A375R
P=0.0004
day
FC of invading cells
A375p A375R
A2058
A2058R
P=0.0024
A2058 A2058R
E
A375p A375R A2058 A2058R
MerTK
pERK
ERK
Akt_pS473
Pan-Akt
pMLC2
Fibronectin
Actin

F

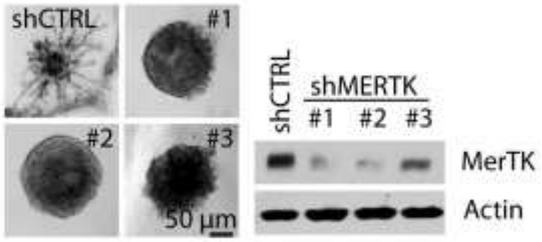

G

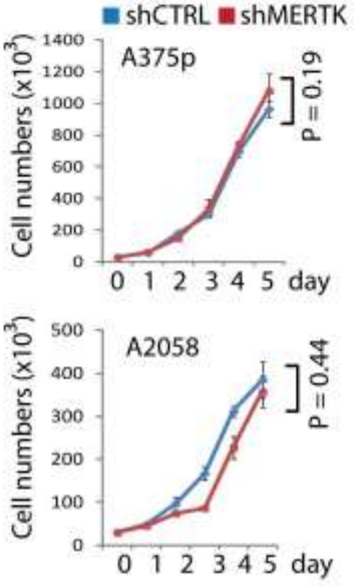

H

Days	12				24			
PLX (µM)	1		2		1		2	
shMERTK	-	+	-	+	-	+	-	+
**A375p**								
colony size	16.1	7.8	9.5	7	446	244.1	416.2	223.1
**A2058**								
colony size	15.6	8.5	8.9	7.4	714.8	462.1	452.8	183.3
**SKMel100**								
colony size	14.9	8.6	7.6	7.8	672.1	321.1	409	219.3

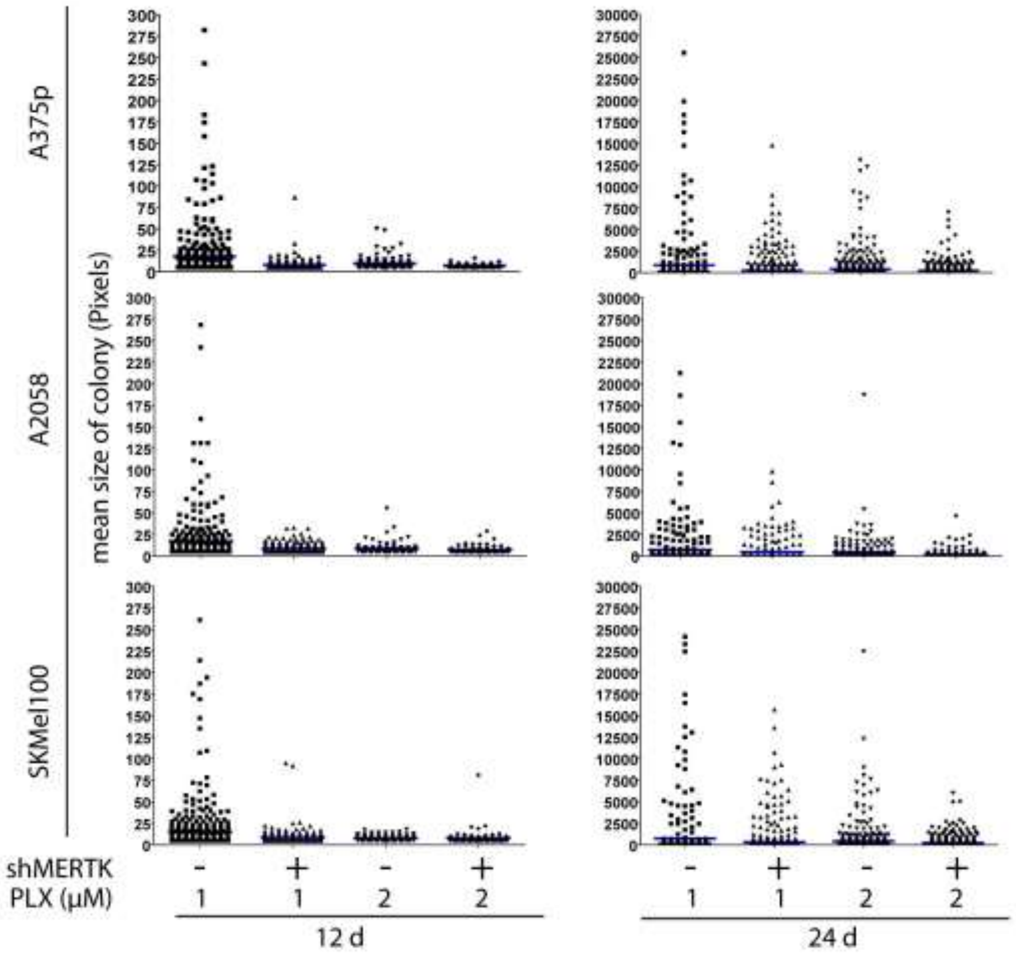

Supplementary Figure 2 A, Phase contrast imaging of morphological changes of A375p and A2058 melanoma cells treated with PLX-4720 for 6 days, and for the established resistant cell lines (A375R and A2058R). The arrows point to filopodia-like membrane protrusions. A375R and A2058R were generated from A375p and A2058 treated with vemurafenib (1 μM) for 2 months. Both cell lines displayed flatter morphology. The MerTK level was determined by western blot (red stars). **B**, Cellular proliferation and invasion assay of A375p and A2058 cells treated with vemurafenib (1 μM) after 3 days. The invasive potential was measured with a standard Boyden-chamber assay. **C,** Comparison of migration patterns between A375p and A375R grown on matrigel. A375p formed clumps, A375R showed an individual migration pattern. **D**, Determination of cellular proliferation and invasion of A375R and A2058R cells. **E**, Western blot analysis of deregulated MLC2 and fibronectin in A375R and A2058R cells. **F**, Evaluation of MERTK shRNAs. Three different sets of shRNA were introduced into A2058. After stable selection, the cells were grown on matrigel. All three sets of shRNA resulted in a similar phenotype. The shRNA that gave the highest knockdown efficacy (#1) was selected for further studies. **G,** Cellular proliferation assay of A375p and A2058 with MERTK knockdown. **H,** Average size of colonies from A375p, A2058 and SKMel100 with MERTK knockdown in presence of 1 or 2 μM of vemurafenib at two given time-points (12 days and 24 days). The size of the colony was reflected by the number of pixels measured with ImageJ.

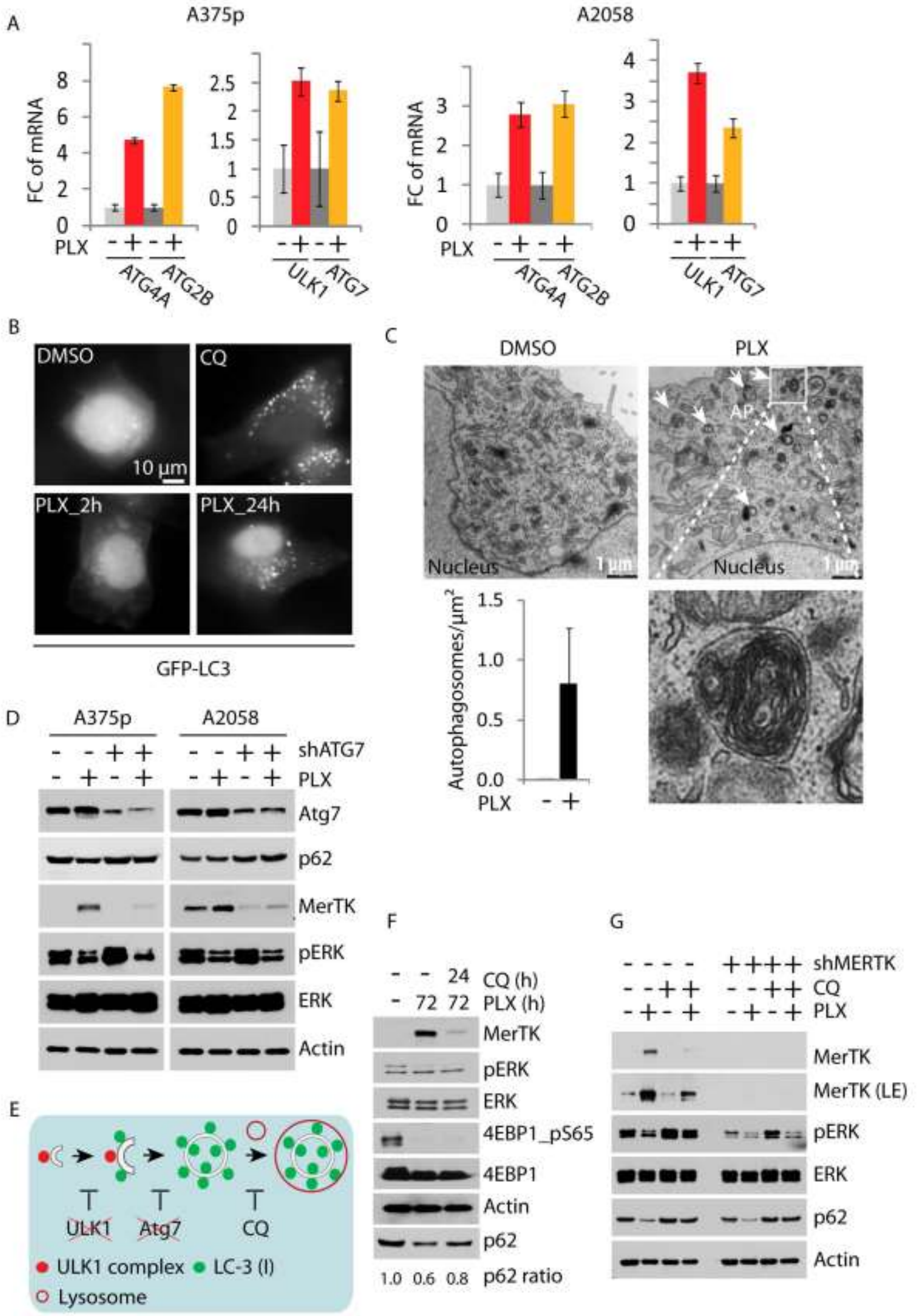
A
A375p
A2058
FC of mRNA
PLX
ATG4A
ATG2B
ULK1
ATG7
B
DMSO
CQ
10 µm
PLX_2h
PLX_24h
GFP-LC3
C
DMSO
PLX
AP
Nucleus
1 µm
Autophagosomes/µm²
D
shATG7
Atg7
p62
MerTK
pERK
ERK
Actin
E
ULK1
Atg7
CQ
ULK1 complex
LC-3 (I)
Lysosome
F
CQ (h)
PLX (h)
4EBP1_pS65
4EBP1
p62 ratio
1.0 0.6 0.8
G
shMERTK
MerTK (LE)

Supplementary Figure 3 A, QPCR analysis of the mRNA level of 4 autophagy-related targets in PLX-4720-treated A375p and A2058 melanoma cell lines (1 μM, 3 days). **B**, Fluorescent imaging of GFP-LC3-expressing A375p cells incubated with PLX-4720 or CQ. Fluorescent puncta indicate labeled autophagosomes. **C,** Endogenous autophagosomes were visualized by transmission electron microscopy in A375p cells treated with vemurafenib (1 μM) for 3 days. White arrows point to the autophagosomes (AP). The AP number was counted independently by two investigators. **D**, Analysis of MerTK expression in A375p and A2058 cells with ATG7 knockdown. **E**, Illustration of targeting autophagy signaling at three different stages. **F**, A375p cells were pre-incubated with PLX-4720 for 48-h and co-incubated with CQ (30 μM) for additional 24 h. MerTK expression and autophagy activity was assessed by measuring anenhanced p62 degradation. **G**, Analysis of PLX-4720-triggered autophagy signaling in A375p cells with stable knockdown of MERTK.

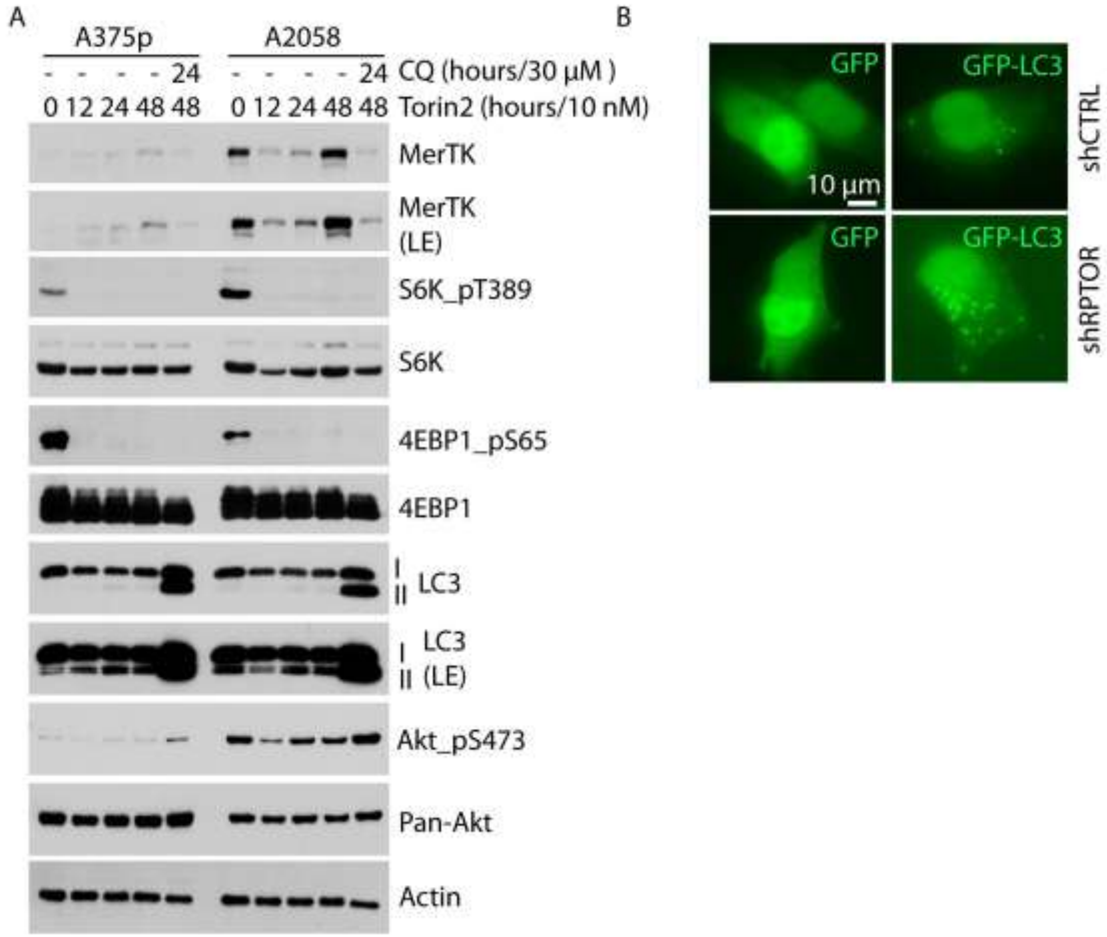

Supplementary Figure 4 A, A375p and A2058 cells were incubated with 10 nM of Torin2. MerTK expression and the activity of autophagy and mTORC1/2 were analyzed at the given time-points. **B**, GFP-LC3 was transiently expressed in A375p cells with RPTOR knockdown for 3 days. Puncta indicate autophagosomes. GFP and parental A375p cells were used as controls.

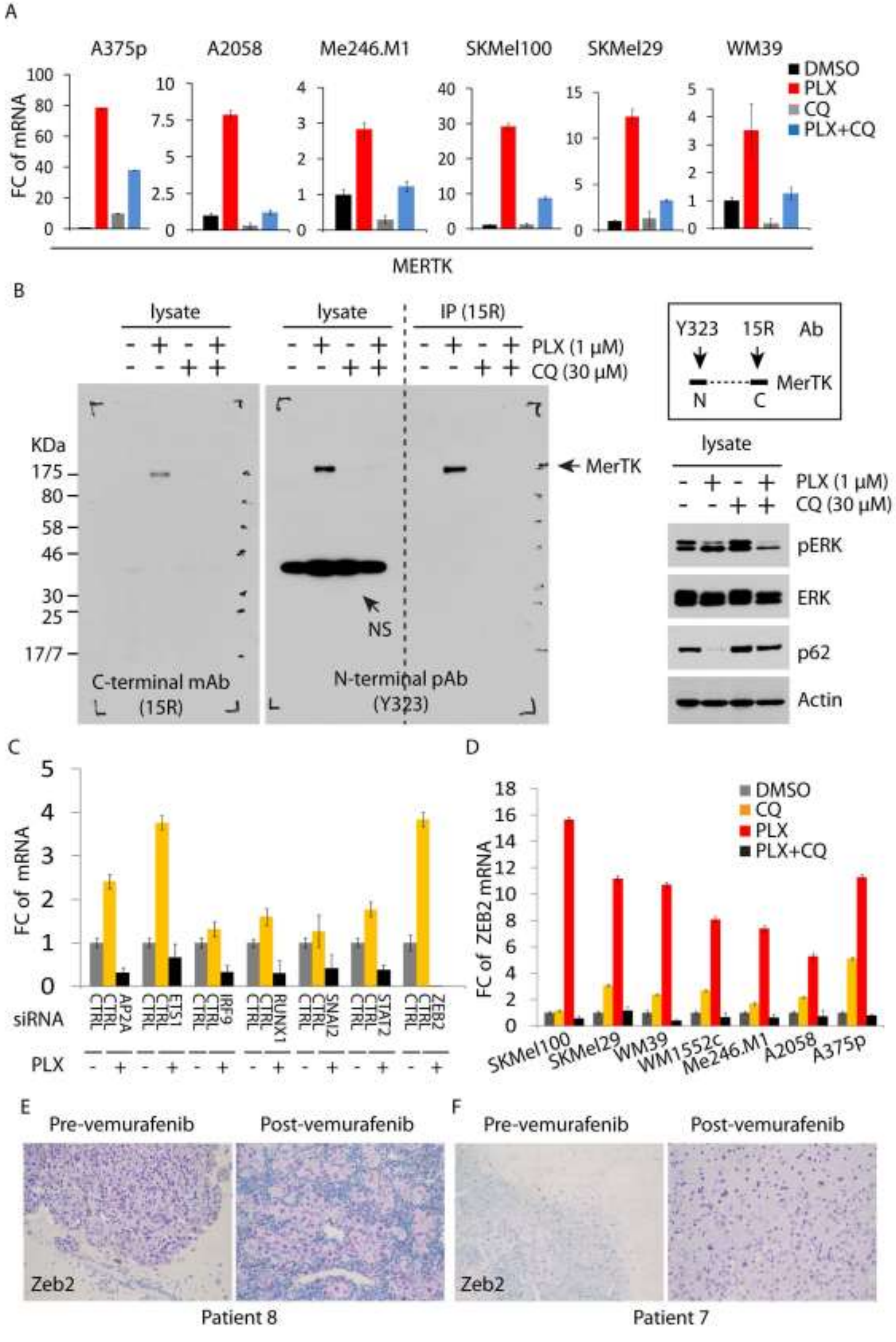
A
A375p
A2058
Me246.M1
SKMel100
SKMel29
WM39
DMSO
PLX
CQ
PLX+CQ
FC of mRNA
MERTK
B
lysate
lysate
IP (15R)
PLX (1 μM)
CQ (30 μM)
Y323
15R
Ab
MerTK
N
C
KDa
MerTK
NS
C-terminal mAb (15R)
N-terminal pAb (Y323)
lysate
PLX (1 μM)
CQ (30 μM)
pERK
ERK
p62
Actin
C
FC of mRNA
siRNA
PLX
CTRL
AP2A
ETS1
IRF9
RUNX1
SNAI2
STAT2
ZEB2
D
DMSO
CQ
PLX
PLX+CQ
FC of ZEB2 mRNA
SKMel100
SKMel29
WM39
WM1552c
Me246.M1
A2058
A375p
E
Pre-vemurafenib
Post-vemurafenib
Zeb2
Patient 8
F
Pre-vemurafenib
Post-vemurafenib
Zeb2
Patient 7

Supplementary Figure 5 A, QPCR analysis of fold change of MERTK mRNA in 6 melanoma cell lines treated with PLX (1 μM), CQ (30 μM) or combo therapy for 2 days. **B,** A375p cells were treated with PLX (1 μM), CQ (30 μM) or combo therapy for 3 days. MerTK level was determined with two specific antibodies that recognize N-terminal (Y323, polyclonal) or C-terminal (15R, monoclonal) epitopes. NS: non-specific. In the IP experiment, endogenous MerTK was immunoprecipitated with 15R in RIPA buffer and probed with Y323. **C**, QPCR analysis of knockdown efficacy of 7 transcription factors. **D**, Assessment of MERTK mRNA level in 7 melanoma cell lines transiently expressing ZEB2 siRNA for 3 days. **E & F**, Paired human melanomas stained for Zeb2.

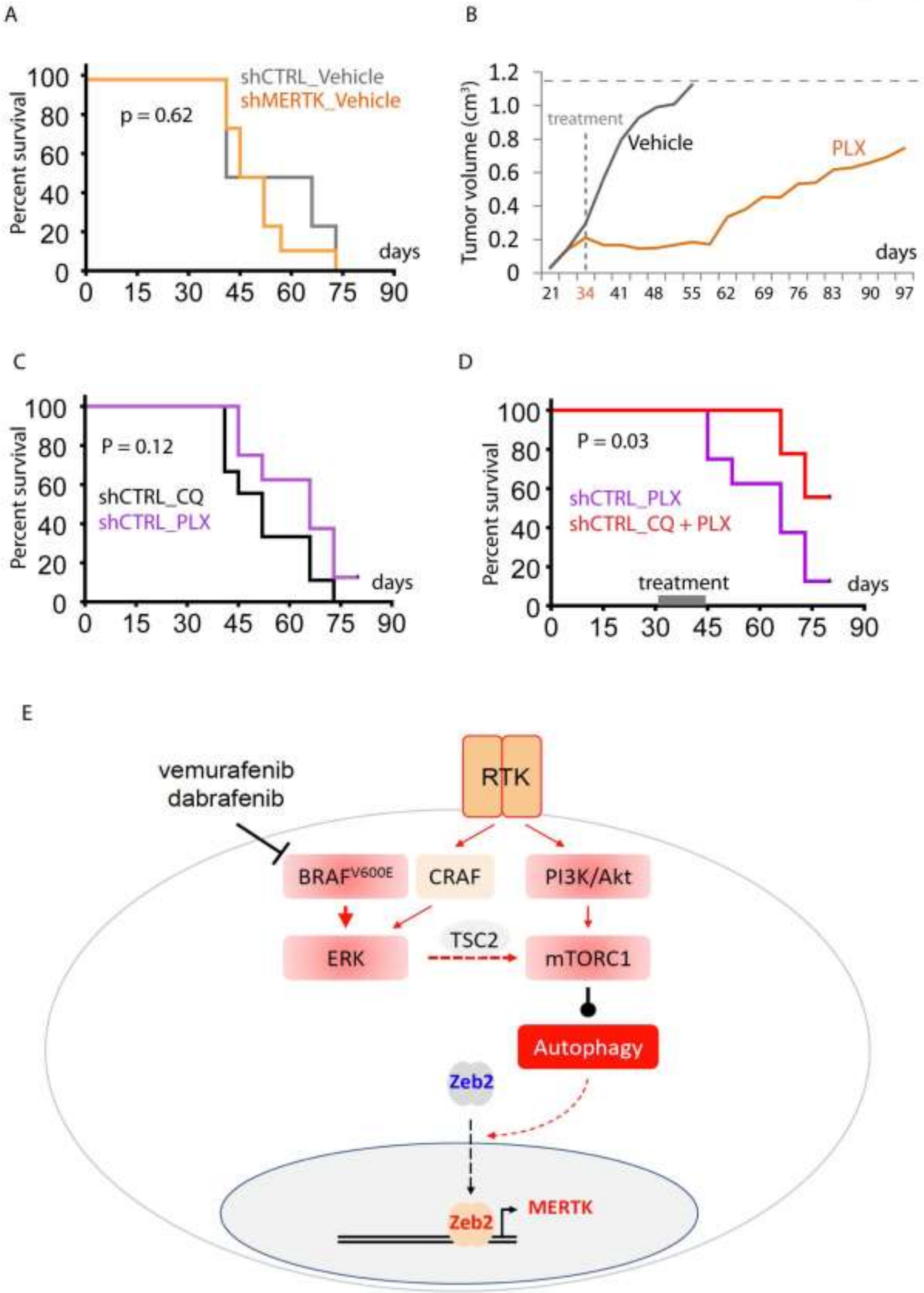
A
100
80
60
40
20
0
Percent survival
shCTRL_Vehicle
shMERTK_Vehicle
p = 0.62
days
0 15 30 45 60 75 90
B
1.2
1.0
0.8
0.6
0.4
0.2
0
Tumor volume (cm³)
treatment
Vehicle
PLX
days
21 34 41 48 55 62 69 76 83 90 97
C
100
80
60
40
20
0
Percent survival
P = 0.12
shCTRL_CQ
shCTRL_PLX
days
0 15 30 45 60 75 90
D
100
80
60
40
20
0
Percent survival
P = 0.03
shCTRL_PLX
shCTRL_CQ + PLX
treatment
days
0 15 30 45 60 75 90
E
vemurafenib
dabrafenib
RTK
BRAFV600E
CRAF
PI3K/Akt
ERK
TSC2
mTORC1
Autophagy
Zeb2
Zeb2
MERTK

Supplementary Figure 6 A, Kaplan-Meier survival analysis of nude mice inoculated with A375p_shCTRL or A375p_shMERTK. The mice were necropsied when the tumour volume reached ~ 1cm^3. Each group contained 15 ~ 18 mice. **B,** Nude mice inoculated with A375p_shCTRL cells were treated with vehicle or PLX (daily). The treatment started on day 34 and stopped on day 97. The tumour size was measured twice a week. Each group contained 10 mice. Till day 55, all vehicle-treated tumours reached the limit of ~ 1 cm^3, whereas the PLX-treated mice showed dramatic repression of tumour growth (tumour volume was ~ 0.2 cm^3) at the exponential growth phase. However, tumors started to regrow from day 62. **C,** Kaplan-Meier survival analysis of mice inoculated with A375p_shCTRL cells in combination with CQ (twice a week) or PLX (daily). **D,** Kaplan-Meier survival analysis of nude mice inoculated with A375p_shCTRL cells and treated with PLX or combo treatment (PLX + CQ). The daily treatment was carried out between day 31 and 45 (acute treatment). The mice were maintained under normal condition for additional 35 days (80 days post-injection in total). Analysis on day 80 was presented with the P value obtained with GraphPad. n = 8~10 mice per group. **E**, Summary of the autophagy-induced MerTK overexpression in metastatic melanomas expressing mutant BRAF. Briefly, BRAFi (vemurafenib or dabrafenib) treatment blocks BRAFV600E/MAPK activity that inhibits mTORC1. This leads to the activation of autophagy, which consequently triggers Zeb2 nuclear translocation. Activated Zeb2 binds to the MERTK promoter and drives its transcription. Upregulated MerTK is overexpressed and localizes on membrane, which in turn may participate in restoration of ERK and Akt phosphorylation and thus confer resistance against BRAFi therapy.

Supplementary Table 1 Sequences of qPCR primers, siRNAs and shRNAs used in this manuscript.

QPCR primers	Target	Forward	Reverse
	MERTK	ACTTCAGCCACCCAAATGTC	GGGCAATATCCACCATGAAC
	AXL	CCCCATATCCGGGCGTGGAGAACA	CTCCTGGGCAGGAGGCAAGGCCTT
	PIK3R1	ACCACTACCGGAATGAATCTCT	GGGATGTGCGGGTATATTCTTC
	CMPK2	GTACCTCCTTTATTCCTGAAGCC	ATGGCAACAACCTGGAACTTT
	ATG4A	TGCTGGTTGGGGATGTATGC	GCGTTGGTATTCTTTGGGTTGT
	ATG2B	AACTGCTGACGAATCCTCAGG	GGGGTTCCAGCTAGGTGAGA
	ATG8	ACTCGCTGGAACACAGATGC	TCTGAGAGCCTGAGACCTTTT
	ATG7	CAGTTTGCCCCTTTTAGTAGTGC	CCAGCCGATACTCGTTCAGC
	ULK1	GGCAAGTTCGAGTTCTCCCG	CGACCTCCAAATCGTGCTTCT
	PROS1	TTGCACTTGTAAACCAGGTTGG	CAGGAACAGTGGTAACTTCCAG
shRNAs	Target	Sequence (5'-3')	Source (ID)
	MERTK #1	ACTTGTGTAGACTCGGTCT (antisense)	Openbiosystems (V3LHS_372202)
	MERTK #2	ATTTCATACAGTTCATCCA (antisense)	Openbiosystems (V3LHS_372203)
	MERTK #3	TTGATGTTGATCTGCACTC (antisense)	Openbiosystems (V2LHS_199668)
	ULK1	CCGGACATCGAGAACGTCACCAAGTCTCGAG ACTTGGTGACGTTCTCGATGTTTTTT	Science. 2011, 331(6016):456-61.
	RPTOR	CCGGGGCTAGTCTGTTTCGAAATTTCTTCCTGT CAAAATTTCGAAACAGACTAGCCTTTTTG	Science. 2005, 307(5712):1098-101.
	RICTOR	CGGGCAGCCTTGAACTGTTTAACTTCCTgTCAT TAAACAGTTCAAGGCTGCTTTTTG	Science. 2005, 307(5712):1098-101.
	ATG7	GGAGTCACAGCTCTTCCTTAC (sense)	Genes Dev. 2009, 23(7): 798–803.
siRNAs	Target	Sense strand sequence (core seq.) 5'-3'	
	RUNX1	AUG GCG CAA CAG CUA UUA A	
	IRF9	UCA AAC AAG UAU ACC AAC A	
	STAT2	AAA GAG AGC UCG CAA CAA A	
	ETS1	GGAGAUGGCUGGGAAUUCAAACUUU	
	SNAI2	AUUUCUUUACAUCAGAAUGGGUCUG	
	AP2A	AAGCAGUAGCUGAAUUUCUCA	
	ZEB2	GCACAACAACGAGAUUCTA	
QPCR primers	Target	Forward	Reverse
(ChIP assay)	MERTK_P1	GCCGGCCGCTTGGCTCCGCCAC	GGCTGCGGATCTGTAATTTC
	MERTK_P2	CCTTCCCCGCCGTCTGCCCAG	GGAGGTGCCGAGGGCTGGAAG
	MERTK_P3	GGACCCTCCTCTTGGGTCTG	AGGGAGCCGGAAAGAGAAGTG
	MERTK_P4	GAGGTGAGCTCCTCGCCGCCAG	GAAAACCCTGGCCGCGCCGC

Supplementary Table 2.1 Pathological scoring of MerTK expression in paired melanomas resistant to BRAFi and BRAFi+MEKi.

Patient ID	Pre-BRAFi	MerTK positivity %	MerTK intensity	H-score
1	1167Z09	0.00	0	0
2	I11-17791	20.00	2	40
3	I12-228	10.00	2	20
4	I12-13745	10.00	3	30
5	5953-11	0.00	0	0
6	I10-21563	2.00	2	4
7	B11-107106/1	0.00	0	0
8	2396	0.00	0	0
Z3	B13-34298	40.00	2	80
Z4	H1122444	0.00	0	0
	**Post-BRAFi**	**MerTK positivity %**	**MerTK intensity**	**H-score**
1	I12-00110	5.00	2	10
2	I13-02120	1.00	2	2
3	I12-24640	45.00	3	135
4	I13-17161	2.00	3	6
5	I13-14594	3.00	2	6
6	I12-25865	15.00	2	30
7	I14-07351	2.00	2	4
8	I13-192971E1	95.00	2	190
Z3	H1417321	85.00	3	255
Z4	HS 1557	0.00	0	0
	**Pre-BRAFi/MEKi**	**MerTK positivity %**	**MerTK intensity**	**H-score**
Z1	H156720	0.00	0	0
Z2	H1419870	0.00	0	0
Z5	H1215676	3.00	2	6
	**Post-BRAFi/MEKi**	**MerTK positivity %**	**MerTK intensity**	**H-score**
Z1	HS15 62/5	3.00	2	6
Z2	H16 10344	6.00	2	12
Z5	H15 27734	5.00	2	10

Supplementary Table 2.2 Clinical characteristics of individual melanoma patients.

ID	Age	Gender	Therapy	AJCC Stage	Best Response	PFS (m)	OS (m)
1	69	M	Vemurafenib	IV	PR	5	17
2	78	F	Vemurafenib	IV	PR	11	18
3	48	F	Vemurafenib	IV	CR	10	42
4	48	F	Dabrafenib	IV	PR	12	27
5	35	F	Vemurafenib	IV	PR	23	42
6	49	M	Vemurafenib	IV	SD	13	32
7	69	M	Vemurafenib	IV	PR	26	54
8	67	F	Vemurafenib	IV	PR	12	18
Z1	40	F	LGX818/MEK162	IV	PD	1	3
Z2	66	F	LGX818/MEK162	IV	PR	9	9
Z3	60	M	LGX818	IV	PR	10	20
Z4	43	F	LGX818	IV	SD	11	31
Z5	52	F	LGX818/MEK162	IV	CR	38	49

PFS: progression free survival; OS: overall survival; PR: partial response; CR: complete response; SD: stable disease; PD: progressive disease.

Reference:

1. Penna E, Orso F, Cimino D, Tenaglia E, Lembo A, Quaglino E, Poliseno L, Haimovic A, Osella-Abate S, De Pitta C, Pinatel E, Stadler MB, Provero P, et al. microRNA-214 contributes to melanoma tumour progression through suppression of TFAP2C. EMBO J. 30:1990-2007.
2. Marone R, Erhart D, Mertz AC, Bohnacker T, Schnell C, Cmiljanovic V, Stauffer F, Garcia-Echeverria C, Giese B, Maira SMWymann MP. Targeting melanoma with dual phosphoinositide 3-kinase/mammalian target of rapamycin inhibitors. Mol Cancer Res. 2009; 7:601-613.
3. Nikolaev SI, Rimoldi D, Iseli C, Valsesia A, Robyr D, Gehrig C, Harshman K, Guipponi M, Bukach O, Zoete V, Michielin O, Muehlethaler K, Speiser D, et al. Exome sequencing identifies recurrent somatic MAP2K1 and MAP2K2 mutations in melanoma. Nat Genet. 44:133-139.
4. Xing F, Persaud Y, Pratilas CA, Taylor BS, Janakiraman M, She QB, Gallardo H, Liu C, Merghoub T, Hefter B, Dolgalev I, Viale A, Heguy A, et al. Concurrent loss of the PTEN and RB1 tumor suppressors attenuates RAF dependence in melanomas harboring (V600E)BRAF. Oncogene. 31:446-457.
5. Sarbassov DD, Guertin DA, Ali SMSabatini DM. Phosphorylation and regulation of Akt/PKB by the rictor-mTOR complex. Science. 2005; 307:1098-1101.
6. Xue G, Restuccia DF, Lan Q, Hynx D, Dirnhofer S, Hess D, Ruegg CHemmings BA. Akt/PKB-mediated phosphorylation of Twist1 promotes tumor metastasis via mediating cross-talk between PI3K/Akt and TGF-beta signaling axes. Cancer Discov. 2:248-259.
7. Yla-Anttila P, Vihinen H, Jokitalo EEskelinen EL. Monitoring autophagy by electron microscopy in Mammalian cells. Methods in enzymology. 2009; 452:143-164.

8. Gautier L, Cope L, Bolstad BMIrizarry RA. affy--analysis of Affymetrix GeneChip data at the probe level. Bioinformatics. 2004; 20:307-315.
9. Smyth G. Limma: linear models for microarray data. In: Gentleman R, Carey V, Dudoit S et al., eds. Bioinformatics and Computational Biology Solutions Using R and Bioconductor. New York: Springer; 2005:397–420.
